# Comparative pangenome analysis of *Enterococcus faecium* and *Enterococcus lactis* provides new insights into the adaptive evolution by horizontal gene acquisitions

**DOI:** 10.1186/s12864-023-09945-7

**Published:** 2024-01-03

**Authors:** Dae Gyu Choi, Ju Hye Baek, Dong Min Han, Shehzad Abid Khan, Che Ok Jeon

**Affiliations:** 1 Department of Life Science, Chung-Ang University, 84, HeukSeok-Ro, Dongjak-Gu, 06974 Seoul, Republic of Korea; 2 Atta‑ur‑Rahman School of Applied Biosciences (ASAB), National University of Sciences and Technology (NUST), 44000 Islamabad, Pakistan

**Keywords:** *Enterococcus faecium*, *Enterococcus lactis*, Pangenome, Adaptive evolution, Horizontal gene transfer, Antibiotic resistance

## Abstract

**Background:**

*Enterococcus faecium* and *E. lactis* are phylogenetically closely related lactic acid bacteria that are ubiquitous in nature and are known to be beneficial or pathogenic. Despite their considerable industrial and clinical importance, comprehensive studies on their evolutionary relationships and genomic, metabolic, and pathogenic traits are still lacking. Therefore, we conducted comparative pangenome analyses using all available dereplicated genomes of these species.

**Results:**

*E. faecium* was divided into two subclades: subclade I, comprising strains derived from humans, animals, and food, and the more recent phylogenetic subclade II, consisting exclusively of human-derived strains. In contrast, *E. lactis* strains, isolated from diverse sources including foods, humans, animals, and the environment, did not display distinct clustering based on their isolation sources. Despite having similar metabolic features, noticeable genomic differences were observed between *E. faecium* subclades I and II, as well as *E. lactis*. Notably, *E. faecium* subclade II strains exhibited significantly larger genome sizes and higher gene counts compared to both *E. faecium* subclade I and *E. lactis* strains. Furthermore, they carried a higher abundance of antibiotic resistance, virulence, bacteriocin, and mobile element genes. Phylogenetic analysis of antibiotic resistance and virulence genes suggests that *E. faecium* subclade II strains likely acquired these genes through horizontal gene transfer, facilitating their effective adaptation in response to antibiotic use in humans.

**Conclusions:**

Our study offers valuable insights into the adaptive evolution of *E. faecium* strains, enabling their survival as pathogens in the human environment through horizontal gene acquisitions.

**Supplementary Information:**

The online version contains supplementary material available at 10.1186/s12864-023-09945-7.

## Background

The genus *Enterococcus* encompasses more than 73 species of Gram-positive, catalase-negative, facultatively anaerobic lactic acid bacteria. These bacteria are widely distributed across various habitats, including the gastrointestinal tracts of humans and animals, plants, soil, water, and fermented foods [1, 2]. Among the *Enterococcus* species, *Enterococcus faecium* has garnered significant attention due to its dual nature, manifesting both beneficial and pathogenic characteristics [1, 3]. Clinically, *E. faecium* strains have emerged as significant nosocomial agents, causing range of hospital-acquired infections such as endocarditis, urinary tract infections, and septicemia [4, 5]. Additionally, *E. faecium* strains isolated from patients with ulcerative colitis have been implicated in promoting colitis [6, 7]. The majority of these *E. faecium* pathogens have been identified as vancomycin-resistant *Enterococci* (VRE), displaying widespread resistance to various antibiotics [8, 9]. Furthermore, they often harbor genes associated with biofilm formation, hemolysin, and invasins, acting as virulence factors that exacerbate their pathogenicity [4, 6].

In contrast, *E. faecium* strains have also been identified as non-pathogenic commensal microbes, prevailing abundantly in healthy infants and contributing positively to both human and animal hosts [10–12]. Specifically, certain *E. faecium* strains have been found to mitigate bacterial pathogenesis by enhancing immune signaling pathways [13, 14], in addition to boosting cancer immunotherapy by promoting the efficacy of checkpoint inhibitors [15]. Some strains of *E. faecium* are even commercially marketed as probiotics, aiming to enhance the well-being of humans or animals [16–18]. Moreover, *E. faecium* strains are often found in fermented dairy products, where they play a significant role in enhancing functionality and refining the quality of the fermentation process [19–22].

*Enterococcus lactis*, which was initially isolated from milk samples, has recently been proposed as a novel species closely related to *E. faecium* [23]. Since then, numerous *E. lactis* strains have been isolated and reported, primarily from fermented foods [24–26]. *E. lactis* strains are generally considered as non-pathogenic and probiotic bacteria. They typically exhibit high susceptibility to antibiotics, lack virulence genes, display negative gelatinase activity, and carry genes encoding for antimicrobial enterocins A, B, and P [27–29]. Therefore, due to their non-pathogenic nature, *E. lactis* strains have received less attention in scientific studies compared to the clinically significant *E. faecium* strains.

The dual nature of *E. faecium* strains, capable of exhibiting either pathogenic or beneficial traits, has prompted studies comparing the genomic and pathological features of both types [3, 6, 7, 11, 30]. Nevertheless, the substantial similarities in 16S rRNA gene sequences and metabolic traits between *E. faecium* and *E. lactis* strains have resulted in the misidentification of numerous *E. lactis* strains as non-pathogenic *E. faecium* strains [31]. In turn, this has made it difficult to accurately investigate the specific attributes of *E. faecium* strains. Moreover, studies on the genomic, phylogenetic, and evolutionary characteristics of *E. faecium* strains have often been limited to specific strains, excluding *E. lactis* strains. This limitation has hindered a comprehensive understanding of the broader genomic and evolutionary relationships among pathogenic and non-pathogenic *E. faecium* strains, as well as *E. lactis* strains. Therefore, in this study, we conducted a comparative pangenome analysis, including genome-based phylogenetic examination, utilizing all available *E. faecium* and *E. lactis* genomes from the public GenBank database. This approach aimed to provide a more comprehensive understanding of their genomic, pathogenic, and evolutionary traits.

## Results

### Collection of dereplicated representative genomes of *E. faecium* and *E. lactis*

All genomes classified as *E. faecium* (2,727 genomes) and *E. lactis* (111 genomes) as of January 2023 were obtained from the GenBank database. Following the exclusion of 13 low-quality *E. faecium* genomes, the remaining 2,825 genomes underwent clustering based on the sequence identities of housekeeping genes. From this analysis, 192 high-quality dereplicated genomes were chose as representative genomes for this study (Table S1). Utilizing average nucleotide identity (ANI) analysis, these representative genomes were separated into two identifiable clades, consisting of 128 *E. faecium* and 64 *E. lactis* genomes (Fig. S1). Notably, all genomes within each respective clade displayed ANI values surpassing 98.3% and 97.8% when compared to the type strains of *E. faecium* (NCTC 7171^ T^) and *E. lactis* (KCTC 21015^ T^). These ANI values significantly exceeded the commonly accepted ANI cutoff value (95–96%) typically employed for delineating prokaryotic species [32]. Our results confirmed that the genomes within the identified clades corresponded to *E. faecium* and *E. lactis* genomes, respectively. Intriguingly, the ANI analysis also unveiled a significant number of *E. lactis* strains that were inaccurately classified as *E. faecium* strains in the GenBank database (as depicted in Fig. S1).

### Phylogenetic features of *E. faecium* and *E. lactis* strains based on 16S rRNA gene and genome sequences

**Fig. 1:**
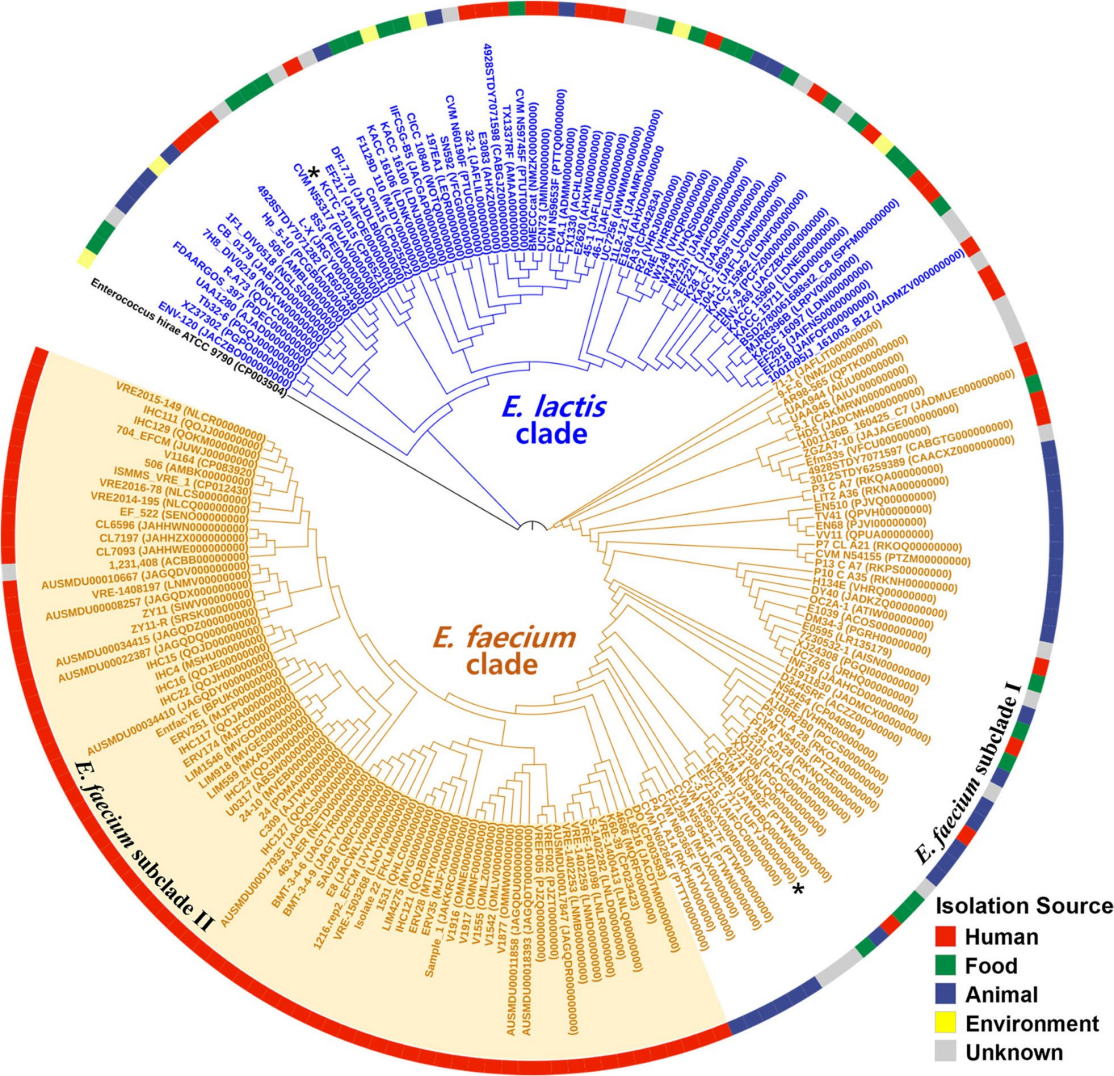
Maximum-likelihood tree showing the phylogenetic relationships between 192 representative genomes of *E. faecium* and *E. lactis*, based on the concatenated sequences of 92 housekeeping genes. *E. hirae* ATCC 9790^ T^ (CP003504) was used as the out-group. The type strains of *E. lactis* and *E. faecium* are marked with an asterisk. The isolation sources of *E. faecium* and *E. lactis* strains are depicted using distinct colors on the outer circle, and the *E. faecium* subclade II, comprising genomes exclusively isolated from humans, is highlighted with a light pink background

Phylogenetic analysis based on 16S rRNA gene sequences revealed that the genomes of *E. faecium* and *E. lactis* did not form distinct clades (Fig. S2). This observation suggests that due to their high sequence similarities, *E. faecium* and *E. lactis* strains cannot be differentiated based on their 16S rRNA gene sequences alone. Therefore, the prevalent misclassification of *E. lactis* strains as *E. faecium* strains in the GenBank database may be attributed to this sequence similarity. However, a distinct separation of *E. faecium* and *E. lactis* genomes into identifiable clades became evident when the analysis was conducted using whole-genome sequences (Fig. 1). This clear differentiation aligns with the observed clustering outcomes from the ANI analysis (Fig. S1).

The collection of genomes from *E. faecium* and *E. lactis* strains isolated from diverse habitats underscores their extensive ecological diversity (Fig. 1). Among the examined *E. faecium* genomes, the majority originated from humans (64.1%) or animals (19.5%), with a smaller proportion originating from foods (5.5%). In contrast, *E. lactis* genomes originated from a broad range of habitats, including foods (31.3%), humans (28.1%), animals (28.1%), and environmental samples (12.5%). Interestingly, a phylogenetic subclade within the *E. faecium* clade, termed *E. faecium* subclade II, which might have diverged more recently, was exclusively composed of genomes originating from humans (except for an unknown strain). This subclade stood apart from other *E. faecium* genomes, which were sourced from diverse environments and categorized as *E. faecium* subclade I (Fig. 1). *E. lactis* genomes exhibited a scattered distribution within the *E. lactis* clade, without forming separate clusters based on their isolation sources. Specifically, *E. lactis* genomes originating from foods, particularly those primarily isolated from traditional fermented foods, displayed a scattered distribution within the *E. lactis* clade. This observation suggests that *E. lactis* strains found in fermented foods might trace their origins back to the diverse environments associated with the fermentation processes of these foods.

**Fig. 2:**
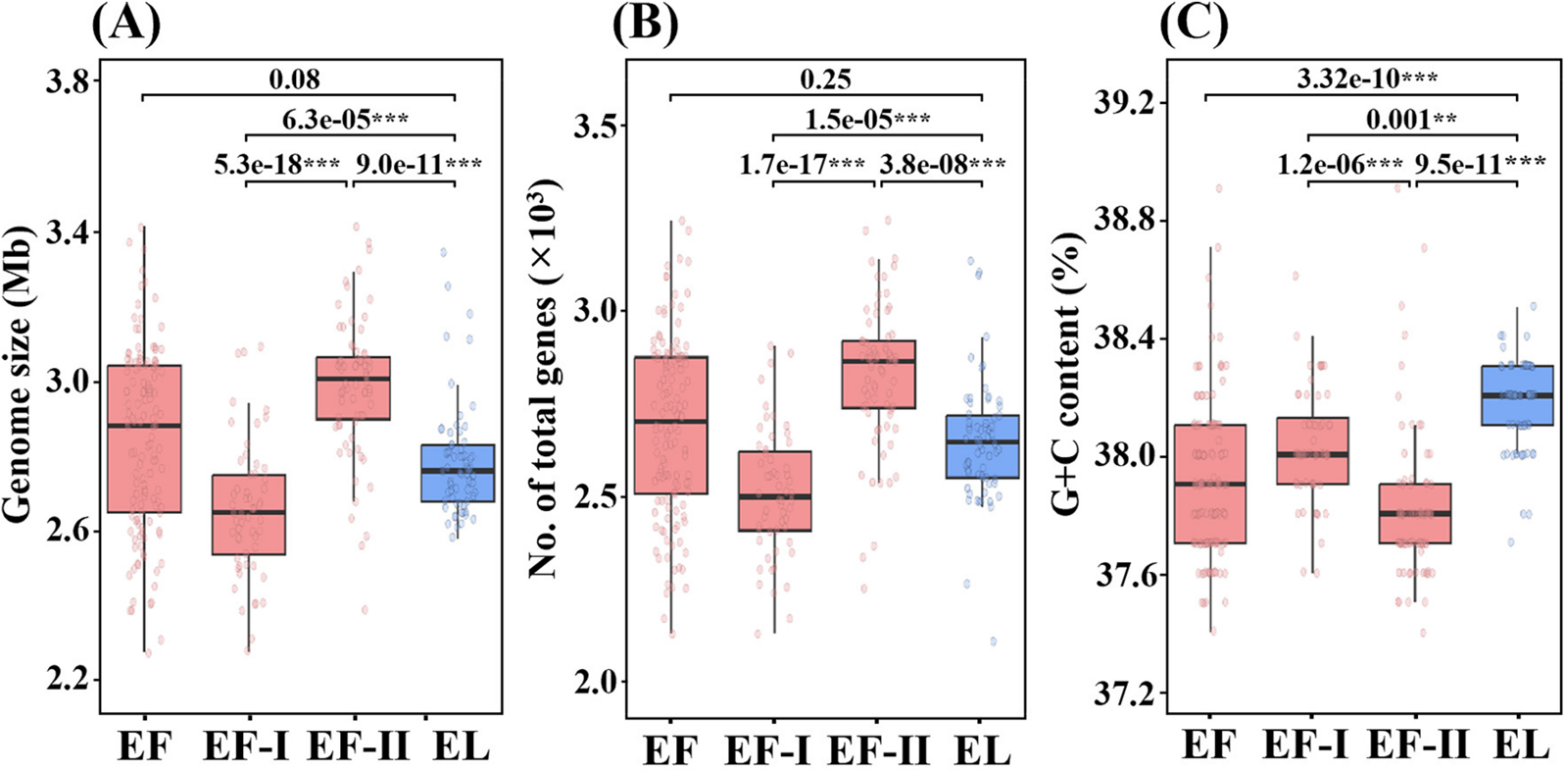
Box plots showing the distributions of sizes (**A**), total gene numbers (**B**), and G + C contents (**C**) of *E. faecium* (subclades I and II) and *E. lactis* genomes. EF, *E. faecium*; EF-I, *E. faecium* subclade I; EF-II, *E. faecium* subclade II; EL, *E. lactis*. *, *p* < 0.01; **, *p* < 0.001; ***, *p* < 0.0001

The sizes and total gene contents of *E. faecium* and *E. lactis* genomes were found to be relatively similar. Specifically, the calculations revealed sizes of 2.85 ± 0.24 Mb and 2,689 ± 239 genes for *E. faecium*, and 2.79 ± 0.15 Mb and 2,649 ± 165 genes for *E. lactis* (Fig. 2). However, notable disparities in genome sizes and gene contents were observed among *E. faecium* subclades I and II, as well as *E. lactis* strains (Figs. 2A and B). Specifically, *E. faecium* subclade II genomes, exclusively derived from humans, exhibited significantly larger genome sizes (2.98 ± 0.20 Mb) and higher gene contents (2,817 ± 200 genes) compared to *E. lactis* genomes and *E. faecium* subclade I genomes, the latter of which exhibited genome sizes of 2.67 ± 0.18 Mb and gene contents of 2,518 ± 170. However, *E. faecium* subclade I genomes had slightly smaller sizes and lower gene content compared to even *E. lactis* genomes. Moreover, the G + C contents of *E. faecium* genomes (37.91 ± 0.26%) were found to be significantly lower than those of *E. lactis* genomes (38.24 ± 0.43%) (Fig. 2C). Particularly, the G + C contents of *E. faecium* subclade II genomes (37.82 ± 0.26%) were significantly lower than those of both *E. faecium* subclade I genomes (38.03 ± 0.19%) and *E. lactis* genomes.

### Metabolic and functional characteristics of *E. faecium* and *E. lactis* strains

To investigate the metabolic features of *E. faecium and E. lactis* strains, we conducted functional analyses based on the Kyoto Encyclopedia of Genes and Genomes (KEGG) database categories using all representative genomes. The overall relative abundances of KEGG functional genes in both species exhibited a high degree of similarity (Fig. S3), suggesting that *E. faecium* strains and *E. lactis* strains may share similar metabolic characteristics. Notably, carbohydrate metabolism and transport-associated genes were highly abundant in both *E. faecium* and *E. lactis* genomes (Fig. S3A), underscoring their versatility in metabolizing various carbohydrates.

Additional insights into the metabolic features of *E. faecium* and *E. lactis* were gained by reconstructing the metabolic pathways for carbon compounds in both species (Fig. S4). The reconstructed metabolic pathways revealed that all *E. faecium* and *E. lactis* strains possess complete glycolysis and 6-phosphogluconate/phosphoketolase pathways with an incomplete tricarboxylic acid cycle, as well as l-lactate dehydrogenase genes, suggesting that both *E. faecium* and *E. lactis* strains participate in both homolactic and heterolactic fermentation, leading to the production of l-lactate, ethanol, acetate, and carbon dioxide as major fermentation products. Moreover, *E. faecium* and *E. lactis* strains were found to be capable of metabolizing a wide range of carbon compounds, including d-glucose, d-fructose, d-galactose, sucrose, maltose, lactose, trehalose, l-arabinose, cellobiose, d-mannose, d-gluconate, d-ribose, raffinose, l-xylulose, galactitol, and d-mannitol, thus highlighting their versatility to adapt metabolically to diverse environments. However, their capacity to metabolize d-sorbitol, d-xylose, and glycerol varied among different *E. faecium* and *E. lactis* strains. The majority of *E. faecium* strains are able to metabolize d-sorbitol and glycerol, whereas only a few *E. lactis* strains possess this capacity. Conversely, the majority of *E. lactis* strains have the ability to metabolize d-xylose, with only a few *E. faecium* strains exhibiting this capability. Moreover, the results of our metabolic pathway analyses revealed that all *E. faecium* and *E. lactis* strains harbor acetolactate synthase (EC 2.2.1.6) and acetolactate decarboxylase (EC 4.1.1.5) genes. These genes are linked to the production of diacetyl and acetoin, both of which are compounds known for contributing distinctive flavors to dairy products, particularly cheese.

Overall, the metabolic profiles of both *E. faecium* and *E. lactis* exhibited a high degree of similarity, without any evident distinguishing metabolic traits. This suggests that these two species share a common phylogenetic ancestor and comparable metabolic capabilities.

### Comparative genomic characteristics of *E. faecium* and *E. lactis* strains

**Fig. 3:**
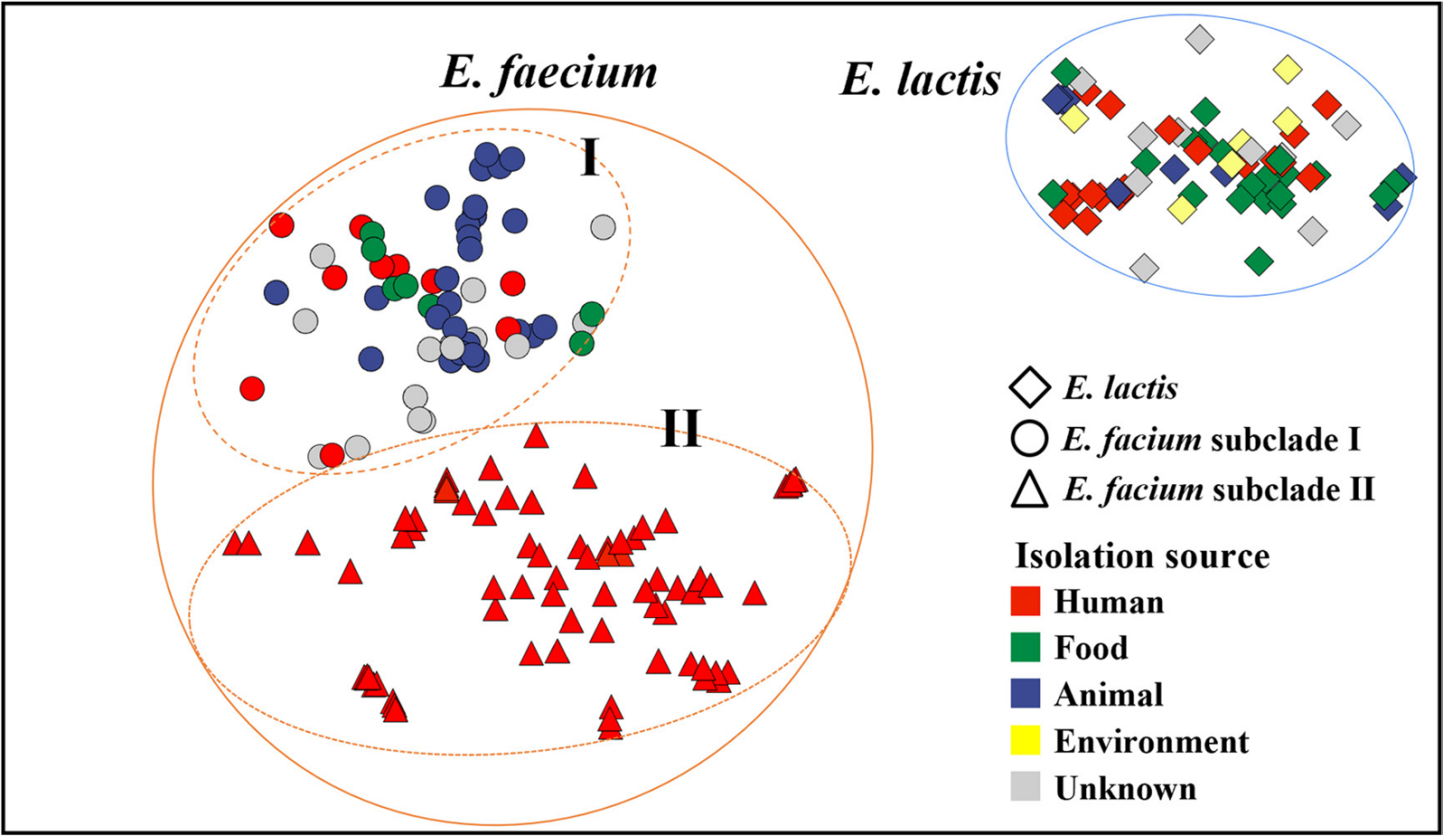
Pangenome neighbor identification for bacterial populations (PANINI) plot of *E. faecium* and *E. lactis* genomes, based on the presence/absence of accessory genes. The taxa and isolation sources of *E. faecium* and *E. lactis* strains (Fig. 1) are indicated using different shapes and colors, respectively

The Pangenome Neighbour Identification for Bacterial Populations (PANINI) analysis, which is based on accessory genes, clearly demonstrated a distinct separation between *E. faecium* and *E. lactis* genomes (Fig. 3). This separation aligns with their species classification (Fig. 1), derived from genome-based phylogenetic analysis. These findings underscore the distinct accessory gene profiles in *E. faecium* and *E. lactis* strains, suggesting the potential existence of independent evolutionary processes involving gene acquisitions and losses, despite their analogous metabolic attributes. Notably, genomes from the human-derived *E. faecium* subclade II formed a distinct cluster that stood apart from *E. faecium* subclade I genomes originating from more varied environments. This separation emphasizes the unique human-adapted features that differentiate *E. faecium* subclade II strains from their *E. faecium* subclade I countparts. However, the PANINI analysis revealed that the accessory gene profiles of *E. faecium* clade I and *E. lactis* genomes were not differentiated based on their isolation sources.

**Fig. 4:**
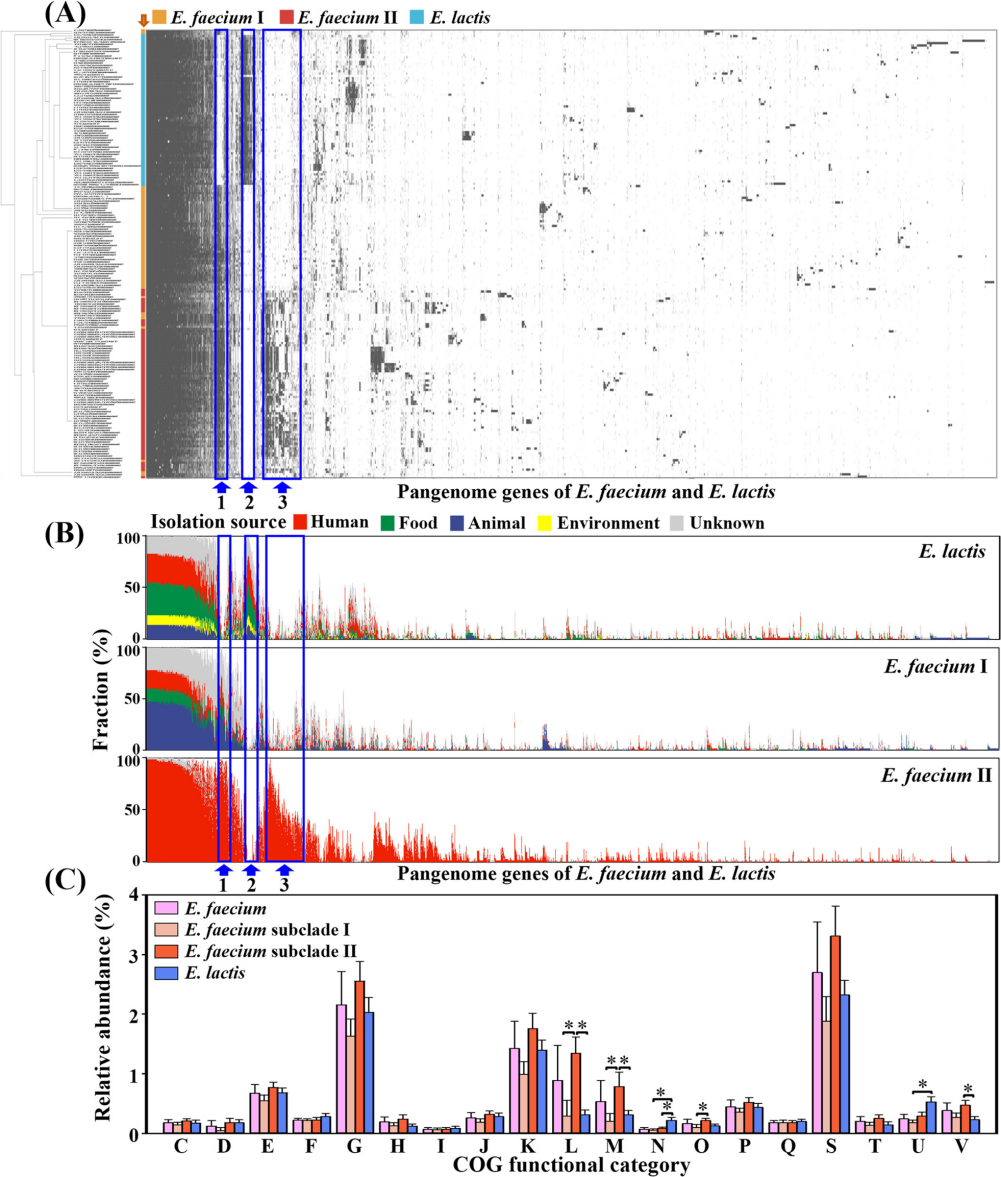
Heatmaps showing the presence (black) or absence (white) of pangenome genes in each *E. faecium* and *E. lactis* genome (**A**) and the fractions (%) of genomes harboring the pangenome genes in the respective *E. faecium* subclades I and II and *E. lactis* (**B**). Hierarchical clustering was performed using the Jaccard distance based on the presence or absence of pangenome genes. Colors indicate the taxonomic classifications (**A**) and isolation sources (**B**) of *Enterococcus* strains. COG classification of genes in regions 1, 2, and 3 (indicated by blue arrows) showing significant differences in the presence or absence of genes between *E. faecium* subclades I and II and *E. lactis* (**C**). The data are expressed as the average relative abundance per genome, with error bars indicating the standard deviations. C, energy production and conversion; D, cell division and chromosome partitioning; E, amino acid transport and metabolism; F, nucleotide transport and metabolism; G, carbohydrate transport and metabolism; H, coenzyme metabolism; I, lipid metabolism; J, translation, ribosomal structure, and biogenesis; K, transcription; L, DNA replication, recombination, and repair; M, cell envelope biogenesis and outer membrane; N, cell motility and secretion; O, post-translational modification, protein turnover, and chaperones; P, inorganic ion transport and metabolism; Q, secondary metabolite biosynthesis, transport, and catabolism; S, function unknown; T, signal transduction mechanisms; U, intracellular trafficking, secretion, and vesicular transport; and V, defense mechanisms. The COG categories that exhibited a high enrichment (more than two times) in *E. faecium* subclade II genomes (or in *E. lactis* genomes) compared to *E. faecium* subclade I genomes and *E. lactis* genomes (or *E. faecium* subclade II genomes) are indicated with an asterisk (*)

Heatmap analyses based on the presence or absence of pangenome genes within the *E. faecium* and *E. lactis* genomes revealed specific gene enrichments in certain clades and deficiencies in others, thus enabling a clear differentiation between *E. faecium* subclades I and II and *E. lactis* strains (Figs. 4A and B). Particularly, genes located in regions 1, 2, and 3 displayed significant differences across *E. faecium* subclades I and II and *E. lactis*. To gain further insights into their functional characteristics, the genes in these regions were functionally classified into Clusters of Orthologous Groups (COG) categories (Fig. 4C). COG category genes associated with cell motility and secretion (N) and intracellular trafficking, secretion, and vesicular transport (U) were found to be enriched in *E. lactis* genomes compared to *E. faecium* subclade I and *E. faecium* subclade II genomes. In contrast, COG category genes involved in DNA replication, recombination, and repair (L), cell envelope biogenesis and outer membrane (M), and defense mechanisms (V) exhibited enrichment in *E. faecium* subclade II genomes relative to *E. faecium* subclade I and *E. lactis* genomes. Particularly, *E. faecium* subclade II genomes showed a significantly higher abundance of COG category genes associated with DNA replication, recombination, and repair (L), including genes involved in horizontal gene transfers (e.g., *tra*, *mob*, and transposase genes) [33].

Further examination of the individual functions of all pangenome genes present in regions 1, 2, and 3 of Fig. 4 was conducted (Table S2). In region 2, the genes enriched in *E. lactis* genomes were related to secretion or transport and included hypothetical functions. Conversely, region 3, characterized by gene enrichment in *E. faecium* subclade II genomes, contained genes associated with antibiotic resistance, virulence factors, bacteriocin synthesis, and mobile elements. These findings provide valuable insights into the clinically relevant genetic characteristics of *E. faecium* subclade II strains within the human environment.

### Abundance and distribution of antibiotic resistance, virulence factor, bacteriocin, and mobile element genes in *E. faecium* subclades I and II and *E. lactis* strains

**Fig. 5:**
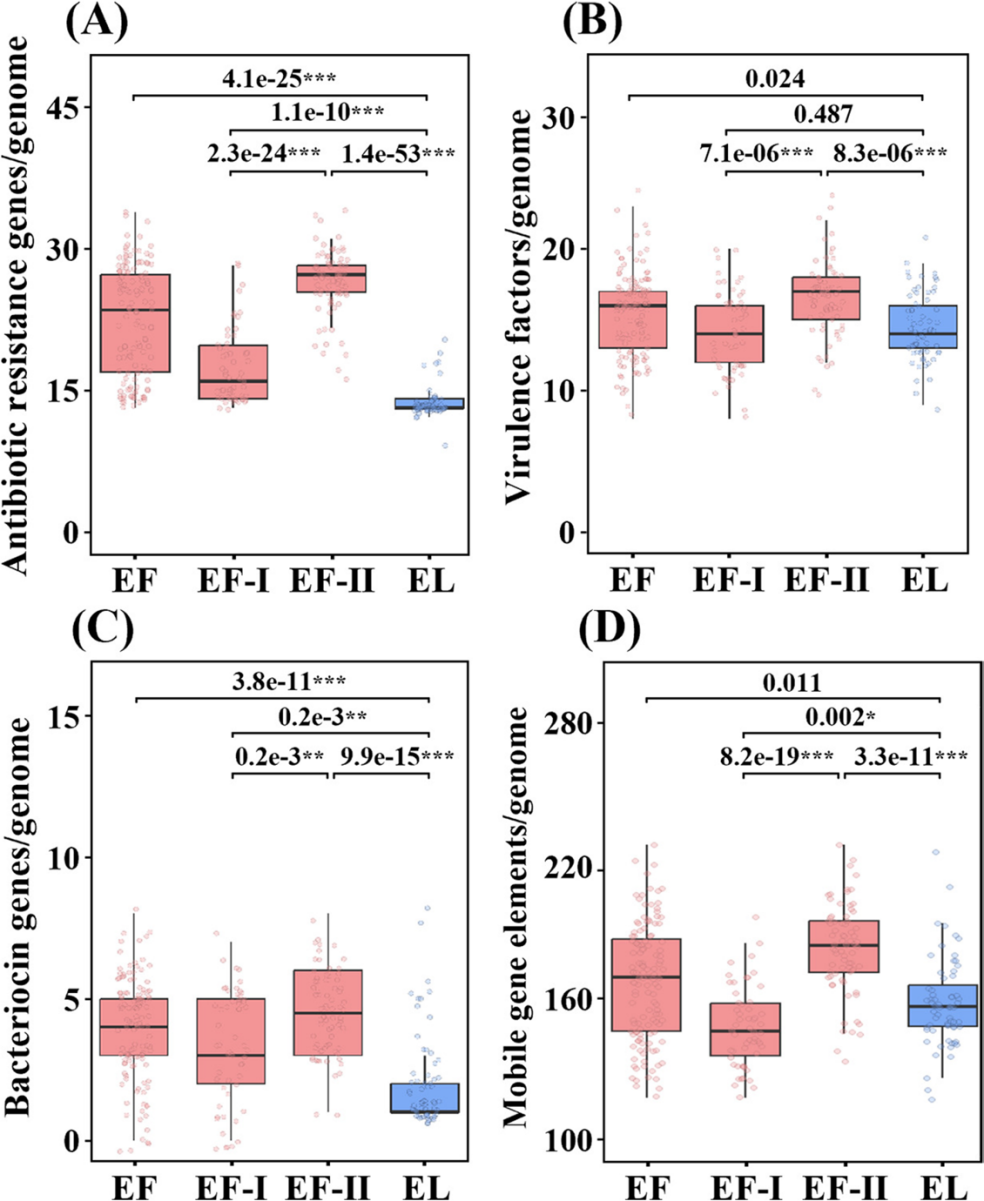
Box plots showing the abundances of genes associated with antibiotic-resistances (**A**), virulence factors (**B**), bacteriocins (**C**), and mobile gene elements (**D**) in *E. faecium* subclades I and II and *E. lactis* genomes. EF, *E. faecium*; EF-I, *E. faecium* subclade I; EF-II, *E. faecium* subclade II; EL, *E. lactis*. *, *p* < 0.01; **, *p* < 0.001; ***, *p* < 0.0001

Statistical analyses were conducted to compare the abundance of antibiotic resistance, virulence factors, and bacteriocin genes among *E. faecium* subclades I and II and *E. lactis* strains, revealing significantly higher abundances of these genes in *E. faecium* strains compared to *E. lactis* strains (Fig. 5). Notably, *E. faecium* subclade II genomes exhibited a significantly greater number of antibiotic resistance genes compared to both *E. faecium* subclade I genomes and *E. lactis* genomes (Fig. 5A). Furthermore, *E. faecium* subclade I genomes, including those derived from animals, also exhibited a higher abundance of antibiotic resistance genes relative to *E. lactis* genomes, which were predominantly sourced from foods.

Regarding virulence factor genes, there was no substantial difference in their prevalence between *E. faecium* genomes, particularly *E. faecium* subclade I genomes, and *E. lactis* genomes (Fig. 5B). However, virulence factor genes in *E. faecium* subclade II genomes were significantly more abundant than in both *E. faecium* subclade I genomes and *E. lactis* genomes, which was consistent with the pattern observed for antibiotic resistance genes. These result might be attributed to the widespread use of antibiotics in humans for the treatment of diseases caused by pathogenic *E. faecium* subclade II strains. In contrast, the application of antibiotics in livestock might not necessarily be linked to the treatment of originating from *E. faecium*. Therefore, even though *E. faecium* subclade I strains are non-pathogenic, they may need to acquire antibiotic resistance genes to survive in antibiotic-treated animal environments.

Concerning bacteriocin genes, their presence was more frequently detected in *E. faecium* genomes compared to *E. lactis* genomes (as presented in Fig. 5C). Notably, *E. faecium* subclade II genomes exhibited significantly higher quantities of bacteriocin genes compared to *E. lactis* genomes. This finding can be attributed to the competitive nature of *E. faecium* strains within the human or animal gut, where they interact with abundant bacterial populations. Notably, the abundance of mobile elements associated with horizontal gene transfers did not exhibit significant disparity between *E. faecium* and *E. lactis* genomes (Fig. 5D). However, *E. faecium* subclade II genomes displayed a markedly higher abundance of mobile elements when compared to both subclade I genomes and *E. lactis* genomes. Notably, transfer genes, including *tra* and *mob* genes, known to be directly related to horizontal gene transfers by conjugation [33], were highly abundant in *E. faecium* subclade II genomes (Fig. S5 and Table S3). These trends suggest that *E. faecium* subclade II strains possess genomic flexibility, making them more prone to acquiring or losing genes through horizontal gene transfers.

**Fig. 6:**
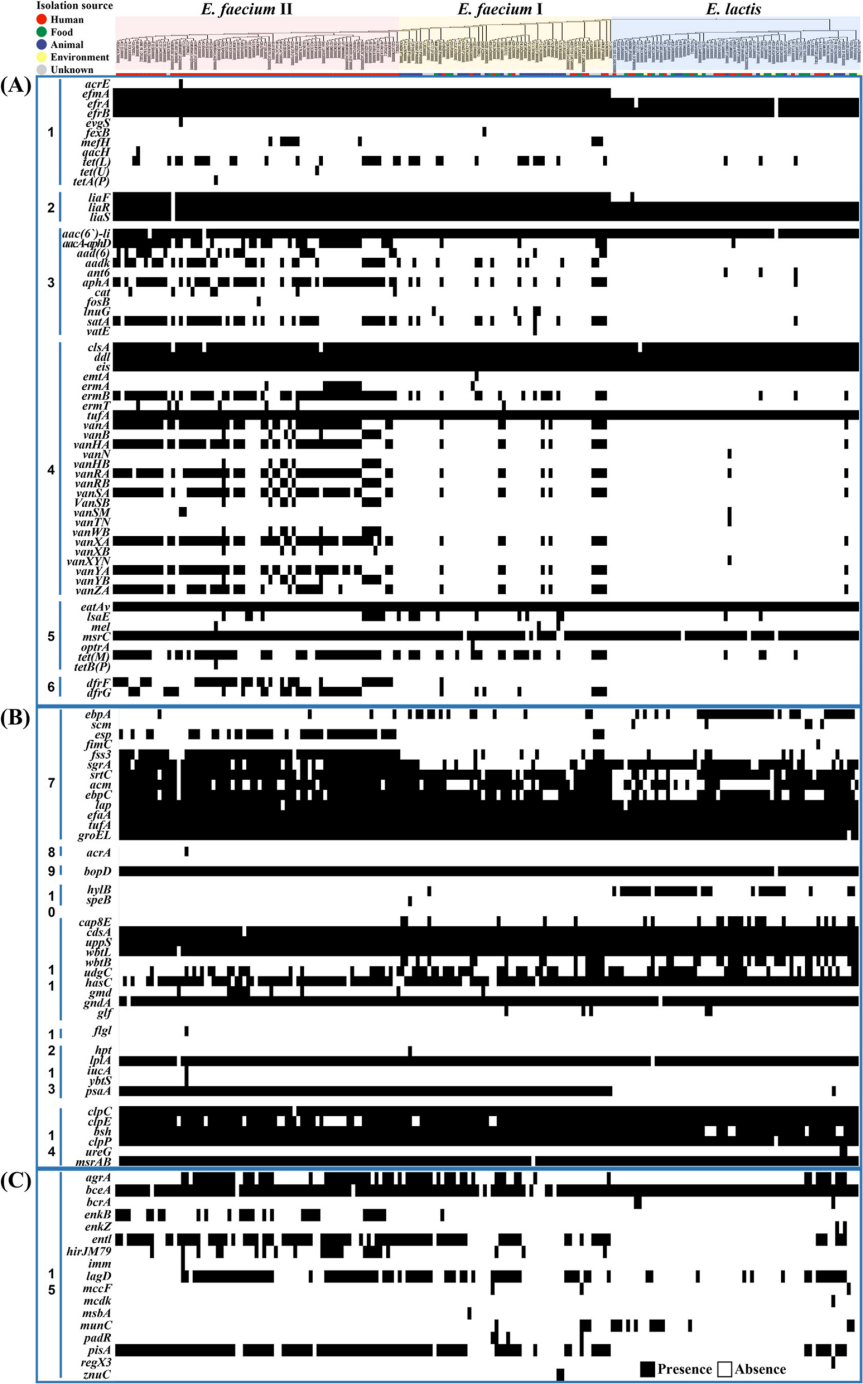
Heatmaps showing the presence (black) or absence (white) of genes associated with antibiotic-resistance (**A**), virulence factors (**B**), and bacteriocin synthesis (**C**) in *E. faecium* subclades I and II and *E. lactis* genomes. The phylogenetic tree of *Enterococcus* genomes based on the 92 housekeeping core genes of the genomes is indicated at the top. 1, Antibiotics efflux (*acrE*, multidrug export protein; *efmA*, multidrug efflux MFS transporter; *efrA,* multidrug efflux ABC transporter subunit; *efrB*, multidrug efflux ABC transporter subunit; *evgS*, sensor protein; *fexB*, aerobic respiration control sensor protein; *mefH*, macrolide-efflux protein; *qacH*, quaternary ammonium compound-resistance protein; *tet(L)*, tetracycline efflux MFS transporter; *tet(U)*, tetracycline resistance ribosomal protection protein; *tetA(P)*, tetracycline efflux MFS transporter; 2, antibiotic efflux; antibiotic target alteration (*liaF*, membrane component associated to the LiaRS two-component system; *liaR,* two-component response regulator; *liaS*, two-component system sensor histidine kinase); 3, antibiotic inactivation (*aac(6’)-li*, aminoglycoside bifunctional resistance protein; *aacA-aphD*, aminoglycoside acetyltransferase; *aad(6)*, putative aryl-alcohol dehydrogenase; *aadk*, aminoglycoside nucleotidyltransferase; *ant6*, aminoglycoside adenyltransferase; *aphA*, aminoglycoside phosphotransferase; *cat*, chloramphenicol acetyltransferase; *fosB*, transcription factor AP-1 subunit; *lnuG*, lincosamide *O*-nucleotidyltransferase; *satA*, streptothricin acetyltransferase; *vatE*, vacuolar H^+^-ATPase E subunit); 4, antibiotic target alteration (*clsA*, cardiolipin synthase A; *ddl*, non van d-Ala-d-Ala ligase; *eis*, *N*-acetyltransferase, kanamycin resistance; *emtA*, rRNA methyltransferase; *ermA,* rRNA adenine *N*-6-methyltransferase*; ermB,* rRNA adenine *N*-6-methyltransferase*; ermT*, rRNA adenine *N*-6-methyltransferase; *tufA*, elongation factor Tu-F; *vanA*, d-Ala-d-lactate ligase; *vanB*, d-Ala-d-lactate ligase; *vanHA*, d-lactate dehydrogenase; *vanN*, d-Ala-d-Ser ligase; *vanH*_*B*_, d-lactate dehydrogenase; *vanRA*, response regulator transcription factor; *vanRB*, response regulator transcription factor; *vanSA*, histidine kinase; *vanSB*, histidine kinase; *vanSM*, histidine kinase; *vanTN*, membrane-bound serine racemase; *vanWB*, glycopeptide resistance accessory protein; *vanXA*, d-Ala-d-Ala dipeptidase; *vanXB*, d-Ala-d-Ala dipeptidase; *vanXYN*, d-Ala-d-Ala dipeptidase/d-Ala-d-Ala carboxypeptidase; *vanYA*, d-Ala-d-Ala carboxypeptidase; *vanYB*, d-Ala-d-Ala carboxypeptidase; *vanZA*, glycopeptide resistance protein) 5, antibiotic target protection (*eatAv*, serine protease; *lsaE*, ABC-F type ribosomal protection protein; *mel*, *α*-galactosidase; *msrC*, ABC-F subfamily protein; *optrA*, ABC transporter ATP-binding protein; *tet(M)*, ribosomal protection protein; *tetB(P)*, tetracycline resistance ribosomal protection protein); 6, antibiotic target replacement (*dfrF,* trimethoprim-resistant dihydrofolate reductase; *dfrG*, trimethoprim-resistant dihydrofolate reductase); 7, adherence (*ebpA,* endocarditis and biofilm-associated pilus subunit; *scm*, collagen adhesin protein; *esp*, surface protein precursor; *fimC*, outer membrane usher protein; *fss3*, collagen binding MSCRAMM; *sgrA*, cell wall anchored protein; *srtC*, fimbrial associated sortase; *acm*, collagen adhesin precursor; *ebpC*, endocarditis and biofilm-associated pilus major subunit endocarditis and biofilm-associated pilus subunit; *lap*, listeria adhesion protein; *efaA*, endocarditis specific antigen; *tufA*, elongation factor Tu-F; *groEL*, chaperonin); 8, antimicrobial activity/competitive advantage (*acrA*, acriflavine resistance protein A); 9, biofilm formation (*bopD*, sugar-binding transcriptional regulator); 10, exoenzyme (*hylB*, hyaluronidase; *speB*, pyrogenic exotoxin); 11, immune modulation (*cap8E*, type 8 capsular polysaccharide synthesis protein; *cdsA*, phosphatidate cytidylyltransferase; *uppS*, undecaprenyl diphosphate synthase; *wbtLB*, glucose-1-phosphate thymidylyltransferase; *ugdC*, UDP-glucose 6-dehydrogenase; *hasC*, UTP-glucose-1-phosphate uridylyltransferase; *gmd*, GDP-mannose 4,6-dehydratase; *gndA*, phosphogluconate dehydrogenase; *glf*, UDP-galactopyranose mutase); 12, motility (*flgl*, flagellar P-ring protein); 13, nutritional/metabolic factor (*hpt*, hexose phosphate transport protein; *lplA*, lipoate protein ligase; *iucA*, aerobactin siderophore biosynthesis protein; *ybtS*, salicylate synthase; *psaA*, manganese ABC transporter); 14, stress survival (*clpC*, endopeptidase Clp ATP-binding chain C; *clpE,* ATP-dependent Clp protease; *bsh*, bile salt hydrolase; *clpP*, ATP-dependent Clp protease proteolytic subunit; *ureG*, urease accessory protein; *msrAB*, methionine sulfoxide reductases); 15, bacteriocin (*agrA*, accessory gene regulator protein A; *bceA*, bacitracin export ATP-binding protein; *bcrA*, bacitracin transport ATP-binding protein; *enkB*, enterocin NKR-5-3B; *enkZ*, enterocin NKR-5-3Z; *entl*, enterolysin A; *hirJM79*, hiracin-JM79; *imm*, colicin immunity protein; *lagD*, lactococcin G transporter; *mccF*, microcin C7 self-immunity protein; *mcdK*, histidine kinase; *msbA*, lipid A-core flippase; *munC*, mundticin KS immunity protein; *padR*, negative transcription regulator; *pisA*, bacteriocin piscicolin-126; *regX3*, sensory transduction protein; *znuC*, zinc import ATP-binding protein)

Subsequent analysis using heatmaps provided a comprehensive overview of the presence or absence of genes associated with antibiotic resistance, virulence factors, and bacteriocin synthesis in each genome, revealing notable variations in their profiles across *E. faecium* subclades I and II and *E. lactis* strains (Fig. 6). Specifically, *E. faecium* subclade II genomes exhibited an abundant presence of antibiotic resistance genes, such as *tet(L)*, *tet(M)*, *vanAHSRYZ(A)*, *aacA-aphD*, *aad(6)*, *aadk*, *aphA*, *satA*, *ermB*, *dfrF*, and *dfrG*, associated with resistance against diverse antibiotics, including tetracycline, vancomycin, aminoglycosides, nucleosides, macrolides, and trimethoprim (Fig. 6A). In contrast, these antibiotic resistance genes were rarely identified in *E. lactis* genomes. Moreover, the *efmA* gene, which encodes a multidrug efflux pump associated with multidrug resistance, was identified in all *E. faecium* genomes but not in all *E. lactis* genomes. These findings strongly suggest that *E. faecium* strains, particularly those belonging to *E. faecium* subclade II, might have acquired these resistance genes as a mechanism to counteract diverse antibiotics commonly used in human medicine, such as tetracycline, vancomycin, aminoglycosides, nucleosides, macrolides, and trimethoprim.

Moreover, the *efrA* and *efrB* (encoding multidrug efflux pumps), *liaR* and *liaS* (associated with daptomycin resistance), *eis* (linked to kanamycin resistance), *eatAv* (conferring resistance to lincosamides, streptogramin A, and pleuromutilins), and *mcrC* (associated with erythromycin and streptogramin B resistance) genes were identified in nearly all *E. faecium* subclades I and II and *E. lactis* genomes. These shared genetic features suggest that both *E. faecium* and *E. lactis* strains may display common resistance to a variety of antibiotics, including daptomycin, kanamycin, lincosamides, pleuromutilins, erythromycin, and streptogramin A and B.

Regarding virulence factors, the genes *esp*, *fss3*, *sgrA* (associated with biofilm formation), and *psaA* (manganese ABC transporter) were more prevalent in *E. faecium* subclade II genomes compared to *E. faecium* subclade I and *E. lactis* genomes (Fig. 6B). However, the *ebpA* and *hylB* genes were more frequently identified in *E. lactis* genomes than *E. faecium* genomes. Particularly, the *hylB* gene was exclusively identified in *E. lactis* genomes, except for a single *E. faecium* subclade I genome. The *ebpA* gene was frequently identified in *E. lactis* genomes, whereas it was only present in a few *E. faecium* subclade II genomes. On the other hand, the *lap*, *efaA*, *bopD* (associated with bacterial adhesion and biofilm formation), *cdsA*, *uppS*, *wbtL*, *hasC*, *gndA*, *clpC*, *clpE*, *bsh*, *clpP*, and *msrAB* (involved in immune modulation and survival under host stress conditions) genes were commonly identified in nearly all *E. faecium* and *E. lactis* genomes. The virulence factor genes identified in the genomes of *E. faecium* subclades I and II and *E. lactis* were found to be associated with the colonization of *Enterococcus* strains in hosts and their subsequent survival within the host (designated as numbers 7 to 14 in Fig. 6). Notably, no genes classified as exotoxins, indicating a direct virulence for hosts, were detected. These results suggest that, while strains within *E. faecium* subclades I and II, as well as *E. lactis*, may exhibit a strong capacity for host invasion and survival within host, they might not directly induce diseases in hosts.

For bacteriocin synthesis genes, the genes *enkB*, *entl*, *hirJM79*, and *pisA*, which are directly involved in bacteriocin production, were more prevalent in *E. faecium* clade II genomes compared to both *E. faecium* clade I genomes and *E. lactis* genomes (Fig. 6C). These findings suggest that *E. faecium* clade II strains may have a greater ability for synthesizing bacteriocins compared to *E. faecium* clade I and *E. lactis* strains, thus potentially enhancing their ability to effectively compete with the high bacterial population in the human gut.

### Principal component analysis (PCA) of antibiotic resistance, virulence factor, and bacteriocin genes, and their phylogenetic characteristics

**Fig. 7:**
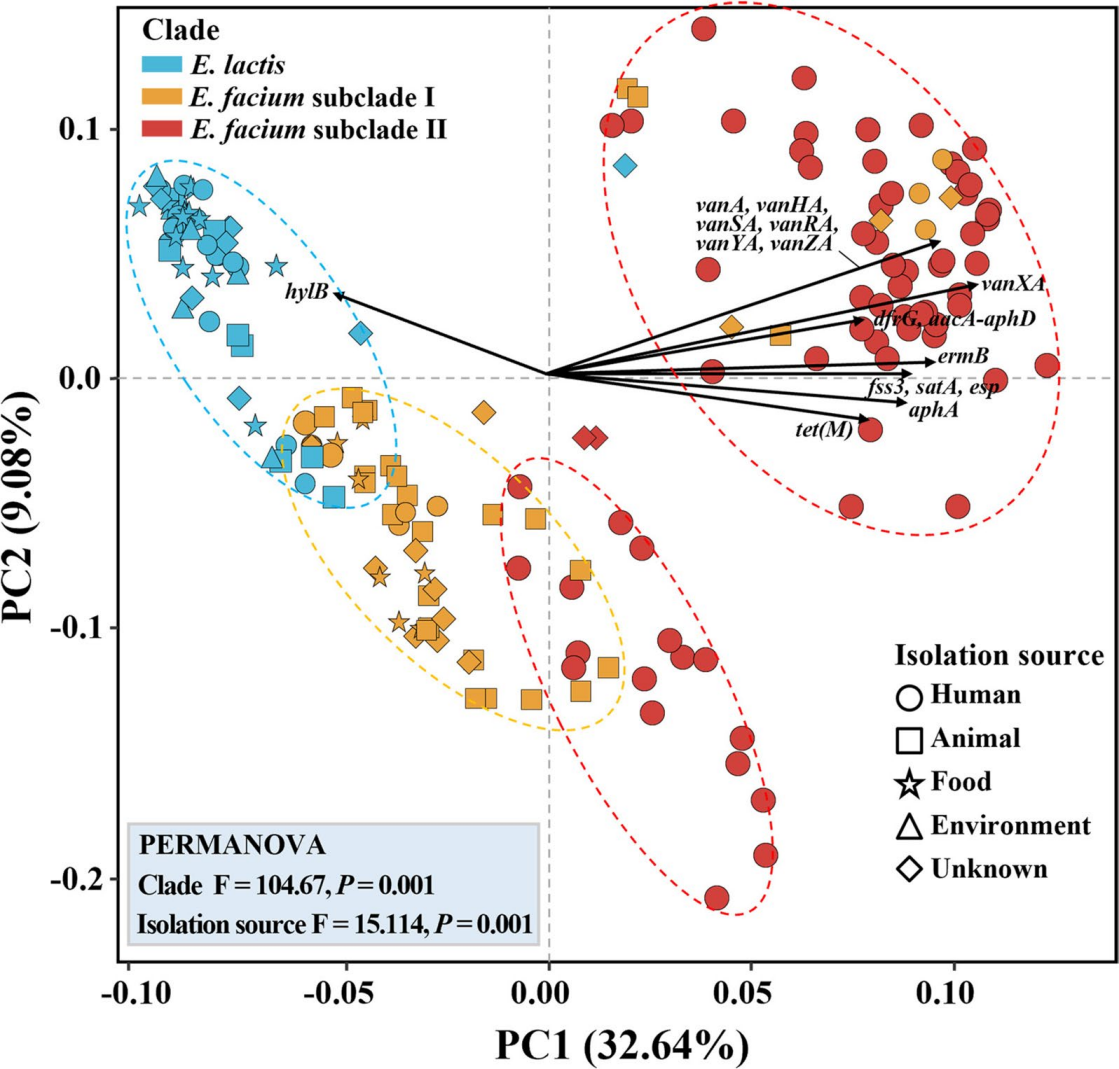
Principal component biplot of *E. faecium* subclades I and II and *E. lactis* genomes, based on the presence or absence of antibiotics resistance, virulence, and bacteriocin genes in their genomes. The arrows represent the relative loading of genes that significantly contribute to the principal components; only genes with contributions exceeding 2% to the principal components are indicated. F and p values in the box represent F statistics and significance of antibiotic resistance, virulence, and bacteriocin gene profiles by clade and isolation source. F-statistic (F) and *p*-values within the box indicate the significance of variations according to *Enterococcus* clades and isolation sources in profiles of antibiotic resistance, virulence, and bacteriocin genes (PERMANOVA)

The PCA results revealed distinct and well-separated clusters of *E. faecium* clades I and II and *E. lactis* genomes based on the presence or absence of antibiotic resistance, virulence, and bacteriocin genes (Fig. 7). Statistical variations in the profiles of antibiotic resistance, virulence, and bacteriocin genes, based on *Enterococcus* clades and isolation sources, were assessed through PERMANOVA analysis. The results demonstrated significant differences in gene profiles according to isolation sources (PERMANOVA R^2^ = 0.2443, F = 15.114, *P* = 0.001) as well as *Enterococcus* clades (PERMANOVA R^2^ = 0.5525, F = 104.67, *P* = 0.001), indicating relatedness between *Enterococcus* clades and isolation sources. Particularly noteworthy was the pronounced profile differentiation observed in *E. faecium* clade II and *E. lactis* genomes (PREMANOVA R^2^ = 0.5943, F = 197.81, *P* = 0.001). The PCA analysis revealed distinct and separate clustering of the majority of *E. faecium* clade II genomes, distinguishing them from both *E. faecium* subclade I and *E. lactis* genomes. However, certain *E. faecium* clade I genomes clustered together with *E. faecium* clade II genomes, and vice versa, suggesting strain-specific variations in antibiotic resistance, virulence, and bacteriocin genes. Importantly, *E. faecium* clade I genomes showed differentiation from *E. faecium* subclade II genomes and clustered more closely with *E. lactis* genomes. This suggests that compared to *E. faecium* clade II strains, *E. faecium* clade I strains share more similar gene profiles with *E. lactis* strains in antibiotic resistance, virulence, and bacteriocin genes.

**Fig. 8:**
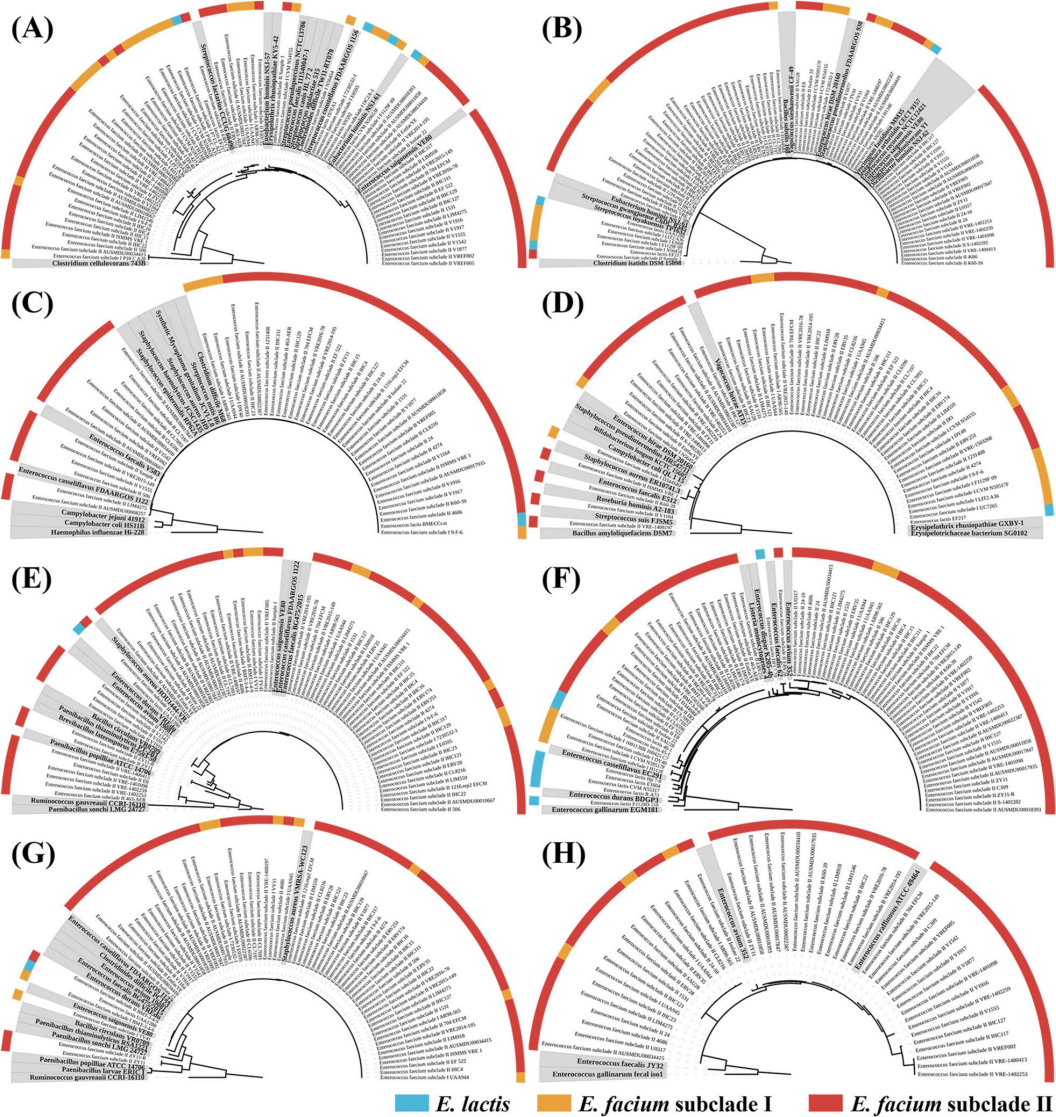
Phylogenetic trees of antibiotic resistance and virulence genes exhibiting significantly different abundances between the genomes of *E. faecium* (subclades I or II) and *E. lactis*. Phylogenetic trees for antibiotic resistance and virulence genes, which were not presented in this figure, can be found in Fig. S6. The trees were constructed using the maximum likelihood algorithm, based on the amino acid sequences. Distinct colors are assigned to *E. faecium* (subclades I or II) and *E. lactis*, corresponding to the source of gene sequences, which are displayed on the outer circle. Closely related GenBank sequences used as references are highlighted with a gray background. **A** *tet(M)*, ribosomal protection protein; **B** *ermB*, rRNA adenine *N*-6-methyltransferase; **C** *aacA-aphD*, aminoglycoside acetyltransferase; **D** *satA*, streptothricin acetyltransferase; **E** *vanXA*, d-Ala-d-Ala dipeptidase; **F** *fss3*, collagen binding MSCRAMM; **G** *vanA*, d-alanine-(R)-lactate ligase; and (**H**) *esp*, surface protein precursor

Biplot analysis revealed that antibiotic resistance genes (*vanAHSRYZ(A)*, *vanXA*, *ermB*, *dfrG*, *aacA-aphD*, *satA*, *tet(M)*, and *aphA*) and virulence factor genes (*fss3*, and *esp*) significantly contributed to the differentiation of major *E. faecium* subclade II genomes from *E. faecium* subclade I and *E. lactis* genomes (Fig. 7). Furthermore, the presence of the *hylB* gene in *E. lactis* genomes significantly contributed to the distinction between *E. faecium* subclade I and II genomes. Phylogenetic analyses were conducted on the genes that displayed significantly differential prevalence in the genomes of *E. faecium* subclade II and *E. lactis* by PCA to infer their putative lateral origin based on phylogenetic distributions or incongruent phylogenetic trees. Our findings revealed that antibiotic resistance and virulence genes (*tet(M)*, *ermB*, *aacA-aphD*, *satA*, *vanXA*, *fss3*, *vanA*, *esp*, and *aphA*) were widely distributed throughout the phylogenetic trees and clustered with genes of other species (Figs. 8 and S5). Particularly, antibiotic resistance genes in *E. faecium* subclade II strains, such as *tet(M)*, *ermB*, *aacA-aphD*, *satA*, and *vanXA* genes (Fig. 8), exhibited a broad distribution across the phylogenetic trees. These findings suggest that *E. faecium* subclade II strains might have independently acquired these genes through horizontal gene transfer. In contrast, all *dfrF* genes in *E. faecium* strains, which confer trimethoprim resistance, exhibited tight clustering and close relatedness to the *dfrF* genes of *Vagococcus teuberi* and *Planococcus plakortidis*, sharing 100% sequence similarities (Fig. S6G). These findings suggest that certain *E. faecium* strains might have acquired *dfrF* genes through horizontal gene transfer from other species, such as *V. teuberi* and *P. plakortidis*, after which they were transferred to other *E. faecium* strains. On the other hand, the *hylB* genes identified in *E. lactis* strains and a single *E. faecium* subclade I strain showed tight clustering with high sequence similarities but were distantly related to the *hylB* genes of other species (Fig. S6H).

## Discussion

The pathogenic or beneficial nature of *E. faecium* strains is subject to variation based on their genetic makeup, and numerous studies have employed phylogenetic and genomic approaches to distinguish between these traits [30, 34]. Galloway-Pena et al. [34] identified two distinct *E. faecium* clades: clade A, mainly comprising clinically relevant isolates, and clade B, primarily consisting of commensal/community-related isolates. Lebreton et al. [30] further subdivided clade A into subclades A1 and A2, with A1 composed mainly of clinical isolates and A2 of animal-relevant isolates. However, a subsequent study questioned the subdivision due to a lack of supporting evidence [35]. Zhong et al. [3] reported genomic variations in *E. faecium* based on isolation sources (e.g., dairy products, hospitals, communities, and animals) and suggested environment-specific genes to distinguish them. While prior studies emphasize the importance of considering origin and clade affiliation, our research indicates that the profiles of antibiotic resistance, virulence genes, genomes, and accessory genes of *E. faecium* clade I and *E. lactis* strains did not clearly exhibit distinctions based on isolation origins (Figs. 1, 3, and 7).

The 16S rRNA gene sequence and metabolic trait similarities between *E. faecium* and *E. lactis* strains suggest potential misclassifications in previous studies characterizing pathogenic and non-pathogenic *E. faecium* strains. Notably, Zhong et al.'s [3] comparative genomic analysis included strains with ANI values below 95%, indicating they were not *E. faecium*. Similarly, Belloso Daza et al. [36] proposed reclassifying strains previously labeled as *E. faecium* clade B as *E. lactis*. Accurate phylogenetic classification of both *E. faecium* and *E. lactis* strains is essential for a comprehensive understanding of genomic characteristics and evolutionary relationships between pathogenic and beneficial strains. Our pangenome analysis revealed no clear clade formations in *E. faecium* and *E. lactis* strains based on 16S rRNA gene sequences (Fig. S2). Metabolic characteristics exhibited a notable resemblance (Figs. S3 and S4). Fig. S1 indicates that many *E. lactis* strains in GenBank are misannotated as *E. faecium*. Nevertheless, our study using 92 housekeeping and accessory genes (Figs. 1 and 3) identified separate phylogenetic clades for *E. faecium* and *E. lactis* strains. Despite similar 16S rRNA gene sequences and metabolic traits, independent speciation events may have led to distinct lineages with different genetic traits. Our phylogenetic analyses suggest *E. faecium*, primarily from humans and animals, may have evolved from *E. lactis*, found in various habitats, including fermented foods (Fig. 1). Notably, *E. faecium* strains exhibit increased antibiotic resistance genes (Fig. 5A), suggesting adaptation to antibiotic use in human and veterinary medicine.

Our genome-based phylogenetic analysis indicates that *E. faecium* subclade II strains, exclusively human-related, may have evolved from *E. faecium* subclade I strains, originating from more diverse environments. *E. faecium* subclade II strains exhibit higher abundances of antibiotic resistance, virulence, bacteriocin synthesis, and mobile element genes than *E. faecium* subclade I and *E. lactis* strains (Figs. 4–6). Additionally, *E. faecium* subclade II strains have larger genomes, higher gene contents, and lower G + C contents than *E. faecium* subclade I and *E. lactis* strains (Fig. 2). These results indicate that *E. faecium* subclade II strains, characterized by extensive genomic plasticity marked by the abundance of mobile elements (Figs. 5D and S5), are likely to acquire genes through frequent horizontal transfers, facilitating their adaptation to human-related environments. This aligns with studies noting the emergence of hospital-associated *E. faecium* strains, particularly vancomycin-resistant strains, coinciding with antibiotic introduction [30].

The enrichment of antibiotic resistance genes, including *tet(L)*, *tet(M)*, *vanAHSRYZ(A)*, *aacA-aphD*, *aad(6)*, *aadk*, *aphA*, *satA*, *ermB*, *dfrF*, and *dfrG*, in *E. faecium* subclade II strains compared to *E. faecium* subclade I and *E. lactis* strains (Figs. 6 and 7), indicates a potential adaptation for survival in human hosts. This adaptation may involve horizontal gene transfers from other species, likely with low G + C contents (Fig. 2C), as an adaptive response to the use of antibiotics in treating pathogenic *E. faecium* subclade II strains. The widespread distribution of these antibiotic resistance genes across the phylogenetic trees supports this observation (Fig. 8). Additionally, the increased presence of virulence factor genes such as *esp*, *fss3*, *sgrA*, and *psaA* in *E. faecium* subclade II (Fig. 6) suggests enhanced adaptability for survival in human hosts, potentially through biofilm formation [37]. In contrast, the reduced presence of virulence factor genes, *ebpA* (associated with pilus formation) and *hylB* (potentially linked to bacterial adhesion and invasion) [38], in *E. faecium* subclade II strains compared to *E. faecium* subclade I and *E. lactis* strains (Fig. 6), suggests potential gene loss during the evolutionary divergence from *E. lactis* to *E. faecium*, particularly in *E. faecium* subclade II. This contrasts with the acquisition of genes through horizontal transfers observed for antibiotic resistance and other virulence factors. The absence of the *hylB* gene, encoding hyaluronidase, in all *E. faecium* subclade II strains further supports this notion. These genes may play a crucial role in the survival of *E. lactis* strains in natural environments, while being dispensable for the survival of *E. faecium* strains in animals or humans.

## Conclusions

This comprehensive pangenome analysis, encompassing all available genomes of *E. faecium* and *E. lactis*, significantly contributes to our understanding of the genomic characteristics and evolutionary relationships of *E. faecium* and *E. lactis* strains. Furthermore, our findings provide valuable insights into various aspects of *E. faecium* and *E. lactis* strains, including their genomic features, pathogenic potential, and evolutionary traits.

## Methods

### Collection of dereplicated representative genomes of *E. faecium* and *E. lactis*

As of February 2023, all publicly available genomes classified as *E. faecium* and *E. lactis* in GenBank were retrieved. The retrieved *E. faecium* and *E. lactis* genomes were subjected to a quality assessment based on measures of completeness and contamination rates, which were evaluated using CheckM [39]. Genomes meeting the criteria of a contamination rate of ≤ 10.0% and a completeness rate of ≥ 90.0%, indicative of the quality of ‘trusted’ genomes [40], were selected for subsequent analyses. To dereplicate the genomes, 92 housekeeping genes were extracted from the high-quality genomes and concatenated using the UBCG2 pipeline [41]. The concatenated genes were then clustered based on a 99.5% nucleotide sequence identity using USEARCH [42]. The genome with the highest completeness in each cluster was chosen as the dereplicated representative genome. To validate the phylogenetic classifications of the representative genomes, ANI values were calculated using a standalone program [43]. The ANI values were graphically represented as heatmaps and subjected to hierarchical clustering using the GENE-E program [44].

### Phylogenetic analyses of *E. faecium* and *E. lactis* genomes based on 16S rRNA gene and genome sequences

To investigate the phylogenetic relationships among the representative genomes of *E. faecium* and *E. lactis*, phylogenetic analyses were conducted using both their 16S rRNA gene and genome sequences. For the 16S rRNA gene-based analysis, 16S rRNA gene sequences were extracted from the representative genomes and aligned, after which a phylogenetic tree was constructed using the maximum-likelihood (ML) algorithm in the MEGA software ver. 7.0 [45]. For the genome-based analysis, the 92 housekeeping gene sequences derived from the representative genomes were concatenated and aligned, and a phylogenetic tree was constructed using the ML algorithm in the UBCG2 pipeline. Both phylogenetic trees were visualized using iTOL [46]. Furthermore, the genome sizes, total gene numbers, and DNA G + C contents of *E. faecium* and *E. lactis* genomes were calculated and visualized as boxplots using the ‘ggplot2’ package (version 4.2.0) in R [47].

### Comparative pangenome analysis of *E. faecium* and *E. lactis*

A comparative pangenome analysis for *E. faecium* and *E. lactis* was performed as described previously [48]. Briefly, the pangenome of the representative genomes was determined using BLASTP with the default cutoff of 95% sequence identity in the Roary pipeline [49]. Functional annotation of pangenome genes was performed using the standalone eggNOG-mapper [50] based on the KEGG database. The annotated genes were categorized using KEGG orthology (KO) numbers, and their abundances in each KEGG category were calculated as percentages of the total gene number in each genome. To examine the metabolic features of *E. faecium* and *E. lactis*, metabolic pathways for various carbohydrates were reconstructed based on predicted KEGG pathways and EC numbers. The absence of metabolic genes in the metabolic pathways was manually confirmed through BLASTP analyses using reference protein sequences corresponding to absent metabolic genes against *E. faecium* and *E. lactis* genomes.

Next, the genomes of *E. faecium* and *E. lactis* were clustered based on accessory genes of the representative genomes using the PANINI tool with the default settings [51]. The presence or absence of pangenome genes in each *E. faecium* and *E. lactis* genome was visualized as heatmaps using the GENE-E program. Hierarchical clustering was performed using "Jaccard distance" and "average linkage" options in the GENE-E program. Pangenome genes in regions showing significant differences between *E. faecium* subclades I and II and *E. lactis* in the presence or absence of genes were assigned to COG categories using eggNOG-mapper, and their relative abundance was calculated as percentages of the total gene number in each genome.

### Abundance and phylogenetic analyses of antibiotic resistance, virulence factor, bacteriocin, and mobile element genes

Antibiotic resistance, virulence factors, antimicrobial element (bacteriocin), and mobile element genes were analyzed by performing BLASTX searches against the comprehensive antibiotic resistance database (CARD) with > 80% identity and > 50% coverage [52], a virulence factor database (VFDB) with > 60% identity and > 60% coverage [53], the BACTIBASE [54] and BAGEL4 [55] databases with 50% identity and > 70%, and the mobileOG-db database [56] with > 90% identity and > 90% coverage, respectively. The abundances of these genes in *E. faecium* and *E. lactis* genomes were visualized as boxplots using the ‘ggplot2’ package in R. The presence of antibiotic resistance, virulence, and bacteriocin genes in each *E. faecium* and *E. lactis* genome was also visualized as heatmaps using GENE-E.

Additionally, PCA of the *E. faecium* and *E. lactis* genomes was conducted using the ‘ggfortify’ and ‘ggplot2’ packages in R according to the presence or absence of antibiotic resistance, virulence, and bacteriocin genes in each genome. The genes exhibiting significant differences in their presence between the genomes of *E. faecium* (subclades I or II) and *E. lactis*, as identified by PCA, were subjected to phylogenetic analyses based on their amino acid sequences using the ML algorithm in MEGA. The most closely related nucleotide sequences with > 50% similarity and > 90% coverage that did not belong to *E. faecium* and *E. lactis* strains in GenBank were used as reference sequences for phylogenetic analyses.

### Statistical analyses

The statistical comparison among *Enterococcus* clades of boxplot data in Figs. 2 and 5 was conducted using Student’s t-test in R [45]. The significance levels were denoted as follows: *p*-values < 0.01 ( ∗), < 0.001 (∗ ∗), or < 0.0001 (∗ ∗ ∗). To assess statistical variations in profiles of antibiotic resistance, virulence factors, and bacteriocin genes across *Enterococcus* clades and isolation sources, a PERMANOVA analysis was conducted using the 'vegan' package in R, utilizing the datasets employed in the PCA analysis.

### Supplementary Information

**Additional file 1.**

**Additional file 2.**

**Additional file 3.**

## Data Availability

The genome datasets analysed for this study are publicly available. The genome data are available here: https://www.ncbi.nlm.nih.gov/datasets/genome/?taxon=1352, and https://www.ncbi.nlm.nih.gov/datasets/genome/?taxon=357441.

## Abbreviations

*E.*: *Enterococcus*
ANI: Average nucleotide identity
KEGG: Kyoto Encyclopedia of Genes and Genomes
PANINI: Pangenome Neighbour Identification for Bacterial Populations
COG: Clusters of Orthologous Groups
PCA: Principal component analysis
ML: Maximum-likelihood
KO: KEGG orthology
CARD: Comprehensive antibiotic resistance database
VFDB: Virulence factor database
