# Supplementary material for: Comparative pangenome analysis of Enterococcus faecium and Enterococcus lactis provides new insights into the adaptive evolution by horizontal gene acquisitions
Source: BMC Genomics. 2024 Jan 3;25:28. doi: 10.1186/s12864-023-09945-7 (PMC10765913; doi:10.1186/s12864-023-09945-7)

**Supplementary Material**

**Table S1** General features^*^ of the representative high-quality genomes of *Enterococcus faecium* and *E. lactis* used for the comparative pangenome analyses in this study. Species names in GenBank are indicated in the table, and the type strains are highlighted in bold.

| Strain name in GenBank (accession no.) | Genome  status^†^  (no. of  contig) | Total  size  (Mb) | G+C  content  (%) | No.  of gene | No. of pseudo-gene | Isolation source |
| --- | --- | --- | --- | --- | --- | --- |
| ***E. faecium*** **NCTC 7171^T^ (UFYJ00000000)** | **D (7)** | **2.67** | **38.1** | **2,595** | **72** | Unknown |
| *E. faecium* V1542 (OMLV00000000) | D (218) | 3.06 | 37.6 | 3,093 | 157 | Rectal |
| *E. faecium* V1917 (OMNF00000000) | D (205) | 3.04 | 37.6 | 3,080 | 147 | Rectal |
| *E. faecium* V1916 (OMNE00000000) | D (222) | 3.05 | 37.6 | 3,095 | 157 | Rectal |
| *E. faecium* V1877 (OMMW00000000) | D (230) | 3.05 | 37.6 | 3,077 | 146 | Rectal |
| *E. faecium* V1555 (OMLZ00000000) | D (234) | 3.05 | 37.5 | 3,082 | 147 | Rectal |
| *E. faecium* E0595 (LR135179) | C (2) | 2.45 | 38.4 | 2,358 | 95 | Unknown |
| *E. faecium* 4928STDY7071597 (CABGTG000000000) | D (133) | 2.67 | 37.9 | 2,734 | 191 | Fecal |
| *E. faecium* 3012STDY6259389 (CAACXZ000000000) | D (9) | 2.71 | 38.2 | 2,685 | 156 | Unknown |
| *E. faecium* 5.1 (CAKMRW000000000) | D (224) | 2.68 | 38 | 2,731 | 176 | Unknown |
| *E. faecium* VRE2015-149 (NLCR00000000) | D (376) | 2.86 | 37.8 | 2,889 | 85 | Perirectal |
| *E. faecium* IHC111 (QOJJ00000000) | D (418) | 2.9 | 37.7 | 2,960 | 114 | Fecal |
| *E. faecium* IHC129 (QOKM00000000) | D (317) | 2.89 | 37.7 | 2,927 | 112 | Fecal |
| *E. faecium* 704_EFCM (JUWJ00000000) | D (608) | 2.84 | 37.7 | 2,863 | 108 | Human |
| *E. faecium* V1164 (CP083920) | C (10) | 2.72 | 38.2 | 2,641 | 73 | Human |
| *E. faecium* 506 (AMBK00000000) | D (263) | 2.8 | 37.8 | 2,809 | 100 | Unknown |
| *E. faecium* ISMMS_VRE_1 (CP012430) | C (6) | 3.13 | 37.8 | 3,091 | 146 | Human blood |
| *E. faecium* VRE2016-78 (NLCS00000000) | D (372) | 2.92 | 37.7 | 2,949 | 106 | Perirectal |
| *E. faecium* VRE2014-195 (NLCQ00000000) | D (516) | 2.98 | 37.7 | 3,090 | 155 | Perirectal |
| *E. faecium* EF_522 (SENO00000000) | D (548) | 3.09 | 37.6 | 3,211 | 187 | Infection |
| *E. faecium* CL6596 (JAHHWN000000000) | D (370) | 2.73 | 37.8 | 2,794 | 146 | Wound secretion |
| *E. faecium* CL7197 (JAHHZX000000000) | D (276) | 2.89 | 37.7 | 2,927 | 127 | Fecal |
| *E. faecium* CL7093 (JAHHWE000000000) | D (169) | 2.82 | 37.8 | 2,783 | 95 | Urine |
| *E. faecium* 1,231,408 (ACBB00000000) | D (379) | 3.08 | 38.3 | 2,939 | 201 | Unknown |
| *E. faecium* AUSMDU00010667 (JAGQDV000000000) | D (4) | 3.06 | 37.9 | 3,022 | 227 | rectal |
| *E. faecium* VRE-1408197 (LNMV00000000) | D (142) | 2.32 | 38.4 | 2,285 | 70 | Fecal |
| *E. faecium* AUSMDU00008257 (JAGQDX000000000) | D (2) | 3.01 | 37.9 | 2,953 | 257 | Human |
| *E. faecium* ZY11 (SIWV00000000) | D (1187) | 3.42 | 38.9 | – | – | Hospital |
| *E. faecium* ZY11-R (SRSK00000000) | D (213) | 3.04 | 37.4 | 3,067 | 91 | Clinical laboratory |
| *E. faecium* AUSMDU00034415 (JAGQDZ000000000) | D (4) | 2.98 | 37.9 | 2,902 | 169 | Human |
| *E. faecium* AUSMDU00022387 (JAGQDQ000000000) | D (6) | 3.06 | 37.7 | 2,990 | 223 | Knee |
| *E. faecium* IHC15 (QOJD00000000) | D (549) | 2.92 | 37.8 | 2,979 | 156 | Blood stream |
| *E. faecium* IHC4 (MSHU00000000) | D (386) | 2.92 | 37.8 | 2,964 | 133 | Blood stream |
| *E. faecium* IHC16 (QOJE00000000) | D (706) | 3.08 | 37.7 | 3,240 | 215 | Blood stream |
| *E. faecium* IHC22 (QOJH00000000) | D (446) | 2.81 | 37.8 | 2,863 | 121 | Blood stream |
| *E. faecium* AUSMDU00034410 (JAGQDY000000000) | D (3) | 3.17 | 37.8 | 3,130 | 288 | Human |
| *E. faecium* EntfacYE (BPUK00000000) | D (171) | 3.06 | 37.5 | 3,066 | 137 | Human |
| *E. faecium* ERV251 (MJFP00000000) | D (356) | 2.99 | 37.7 | 2,981 | 96 | Osteomyelitis |
| *E. faecium* IHC117 (QOJA00000000) | D (522) | 3.18 | 37.8 | 3,291 | 153 | Fecal |
| *E. faecium* ERV174 (MJFC00000000) | D (685) | 2.98 | 37.9 | 3,071 | 125 | Peritoneal fluid |
| *E. faecium* LIM559 (MXAS00000000) | D (676) | 2.57 | 38.4 | 2,614 | 122 | Fecal |
| *E. faecium* IHC23 (QOJI00000000) | D (451) | 2.98 | 37.7 | 3,020 | 101 | Blood stream |
| *E. faecium* LIM1546 (MVGO00000000) | D (1) | 2.6 | 38.1 | 2,566 | 150 | Rectal |
| *E. faecium* LIM918 (MVGE00000000) | D (764) | 2.82 | 38.2 | 2,844 | 155 | Fecal |
| *E. faecium* U0317 (ABSW00000000) | D (227) | 2.89 | 37.7 | 2,891 | 117 | Urinary tract infection |
| *E. faecium* 24-10 (MVEB00000000) | D (191) | 2.98 | 37.6 | 3,274 | 261 | Infected wound |
| *E. faecium* 24 (PDMA00000000) | D (197) | 2.95 | 37.8 | 3,021 | 119 | Fecal |
| *E. faecium* C309 (AJTW00000000) | D (199) | 3.1 | 37.6 | 3,170 | 214 | Peritoneal drainage fluids |
| *E. faecium* IHC127 (QOKL00000000) | D (345) | 3 | 37.5 | 3,043 | 103 | Fecal |
| *E. faecium* AUSMDU00017935 (JAGQDS000000000) | D (3) | 3.21 | 37.8 | 3,135 | 226 | Rectal |
| *E. faecium* 463-AER (NETD00000000) | D (9) | 3.15 | 38.1 | 3,121 | 180 | Blood |
| *E. faecium* BMT-3-4-6 (JAGTYR000000000) | D (411) | 2.76 | 38.3 | 2,824 | 120 | Rectal |
| *E. faecium* BMT-3-4-9 (JAGTYO000000000) | D (779) | 2.64 | 38.5 | 2,827 | 192 | Rectal |
| *E. faecium* SAU28 (QBIC00000000) | D (400) | 3.05 | 37.8 | 3,146 | 135 | Human |
| *E. faecium* E8 (JACWLV000000000) | D (222) | 3.02 | 37.8 | 3,065 | 86 | Surface patient room |
| *E. faecium* 1216.rep2_EFCM (JVYK00000000) | D (696) | 2.96 | 37.7 | 3,129 | 160 | Human |
| *E. faecium* VRE-1503268 (LNOY00000000) | D (164) | 2.4 | 38.7 | 2,389 | 76 | Fecal |
| *E. faecium* Isolate 22 (FKLM00000000) | D (320) | 3.38 | 37.5 | 3,554 | 199 | Blood culture |
| *E. faecium* 1531 (QHLC00000000) | D (433) | 3.05 | 37.6 | 3,093 | 145 | Swab |
| *E. faecium* LIM4275 (MVGI00000000) | D (944) | 2.96 | 37.8 | 3,181 | 198 | Fecal |
| *E. faecium* IHC121 (QOJB00000000) | D (291) | 3.1 | 37.7 | 3,143 | 103 | Fecal |
| *E. faecium* ERV28 (MTRY00000000) | D (921) | 3 | 38.3 | 3,201 | 191 | Surgical wound |
| *E. faecium* ERV35 (MJFX00000000) | D (736) | 3.07 | 38.1 | 3,183 | 133 | Blood |
| *E. faecium* Sample_1 (JAKKRC000000000) | D (498) | 2.83 | 38 | 3,045 | 232 | Blood |
| *E. faecium* AUSMDU00011858 (JAGQDU000000000) | D (4) | 3.36 | 37.7 | 3,334 | 269 | Rectal |
| *E. faecium* AUSMDU00018393 (JAGQDT000000000) | D (5) | 3.27 | 37.7 | 3,225 | 242 | Rectal |
| *E. faecium* VREF005 (PJZQ00000000) | D (190) | 2.98 | 37.6 | 2,958 | 209 | Hospital |
| *E. faecium* VREF002 (PJZT00000000) | D (185) | 2.99 | 37.6 | 2,973 | 204 | Hospital |
| *E. faecium* AUSMDU00017847 (JAGQDR000000000) | D (4) | 3.3 | 37.7 | 3,244 | 222 | Swab |
| *E. faecium* VRE-1402253 (LNMB00000000) | D (361) | 3.06 | 38 | 3,203 | 205 | Fecal |
| *E. faecium* VRE-1402259 (LNMD00000000) | D (276) | 3.08 | 37.9 | 3,243 | 227 | Fecal |
| *E. faecium* VRE-1401098 (LNLR00000000) | D (240) | 3.06 | 37.9 | 3,147 | 172 | Fecal |
| *E. faecium* S-1402282 (LNLD00000000) | D (229) | 3.15 | 37.8 | 3,277 | 166 | Fecal |
| *E. faecium* VRE-1400413 (LNLQ00000000) | D (312) | 3.04 | 38 | 3,146 | 188 | Fecal |
| *E. faecium* K60-39 (CP023423) | C (5) | 2.74 | 38 | 2,654 | 109 | Blood |
| *E. faecium* 4686 (MQRF00000000) | D (207) | 3.23 | 37.4 | 3,320 | 163 | Blood |
| *E. faecium* CL8216 (JACDTM000000000) | D (340) | 2.91 | 37.7 | 2,974 | 187 | Fecal |
| *E. faecium* DO (CP003583.1) | C (4) | 2.7 | 38.2 | 2,622 | 98 | Human |
| *E. faecium* CVM N60284F (PTTT00000000) | D (324) | 2.71 | 37.7 | 2,742 | 126 | Sow |
| *E. faecium* P4 CL A14 (RKPH00000000) | D (27) | 2.6 | 37.9 | 2,556 | 55 | Cloaca |
| *E. faecium* CVM N60365F (PTVV00000000) | D (147) | 2.79 | 37.6 | 2,791 | 90 | Beef Cows |
| *E. faecium* F1129F 09 (MJDX00000000) | D (111) | 2.78 | 37.9 | 2,777 | 108 | Fecal |
| *E. faecium* CVM N59542F (PTWN00000000) | D (671) | 2.91 | 37.8 | 3,048 | 174 | Heifers |
| *E. faecium* CVM N59517F (PTWP00000000) | D (732) | 3.1 | 37.6 | 3,361 | 167 | Sow |
| *E. faecium* L-3 (JRGX00000000) | D (68) | 2.64 | 38 | 2,561 | 168 | Probiotic preparation |
| *E. faecium* EF215 (JAIFOC000000000) | D (640) | 3.09 | 37.8 | 3,068 | 148 | Probiotic |
| *E. faecium* CVM N59492F (PTWW00000000) | D (142) | 2.65 | 38 | 2,631 | 98 | Swine |
| *E. faecium* 4274 (QNUQ00000000) | D (367) | 2.69 | 37.8 | 2,675 | 151 | Nasal fossa |
| *E. faecium* P18 C A28 (RKNQ00000000) | D (32) | 2.55 | 38.2 | 2,478 | 52 | Ceaca |
| *E. faecium* CVM N54035 (PTZE00000000) | D (130) | 2.7 | 37.8 | 2,689 | 87 | Chicken breast |
| *E. faecium* P5 CL A 28 (RKOA00000000) | D (216) | 2.72 | 38 | 2,727 | 123 | Cloaca |
| *E. faecium* XJ1306 (PGQK00000000) | D (93) | 2.76 | 38 | 2,689 | 114 | Yoghurt |
| *E. faecium* IQ110 (LKPG00000000) | D (44) | 2.8 | 37.9 | 2,793 | 91 | Cheese |
| *E. faecium* 1,231,501 (ACAY00000000) | D (140) | 2.87 | 38.2 | 2,770 | 83 | Unknown |
| *E. faecium* A108R2B0 (PGCS00000000) | D (308) | 2.95 | 38 | 2,973 | 123 | Rectum |
| *E. faecium* H112E (VHRR00000000) | D (42) | 2.66 | 38 | 2,592 | 122 | Beef carcass |
| *E. faecium* N56454 (CP040904) | C (2) | 2.5 | 38.2 | 2,378 | 98 | Retail chicken |
| *E. faecium* D344SRF (ACZZ00000000) | D (215) | 2.75 | 37.9 | 2,796 | 167 | Unknown |
| *E. faecium* U1911830 (JADMCX000000000) | D (51) | 2.42 | 38.1 | 2,368 | 138 | Unknown |
| *E. faecium* INF39 (JAAHCD000000000) | D (625) | 2.64 | 38.6 | 2,690 | 209 | Brine cheese |
| *E. faecium* UC7265 (JRHQ00000000) | D (703) | 2.81 | 37.8 | 2,901 | 228 | Human |
| *E. faecium* XJ24308 (PGQI00000000) | D (105) | 2.52 | 38.1 | 2,466 | 164 | Yoghurt |
| *E. faecium* 7230532-1 (AISN00000000) | D (33) | 2.59 | 38.3 | 2,477 | 99 | Pig |
| *E. faecium* DM34-3 (PGRH00000000) | D (181) | 2.66 | 38 | 2,650 | 175 | Yoghurt |
| *E. faecium* E1039 (ACOS00000000) | D (124) | 2.5 | 38 | 2,516 | 136 | Human |
| *E. faecium* OC2A-1 (ATIW00000000) | D (119) | 2.68 | 37.9 | 2,612 | 85 | Unknown |
| *E. faecium* DY40 (JADKZQ000000000) | D (145) | 2.61 | 38 | 2,567 | 166 | Swine |
| *E. faecium* H134E (VHRQ00000000) | D (66) | 2.51 | 38.1 | 2,434 | 78 | Beef carcass |
| *E. faecium* P10 C A35 (RKNH00000000) | D (108) | 2.29 | 38.3 | 2,304 | 170 | Ceaca |
| *E. faecium* P13 C A7 (RKPS00000000) | D (92) | 2.42 | 38.3 | 2,371 | 105 | Ceaca |
| *E. faecium* CVM N54155 (PTZM00000000) | D (257) | 2.62 | 37.8 | 2,630 | 133 | Ground turkey |
| *E. faecium* P7 CL A21 (RKOQ00000000) | D (113) | 2.4 | 38.3 | 2,368 | 132 | Cloaca |
| *E. faecium* VV11 (QPUA00000000) | D (218) | 2.52 | 38 | 2,483 | 167 | Poultry fecal |
| *E. faecium* EN68 (PJVI00000000) | D (218) | 2.49 | 38.1 | 2,427 | 162 | Chicken carcass |
| *E. faecium* TV41 (QPVH00000000) | D (224) | 2.54 | 38 | 2,500 | 181 | Poultry fecal |
| *E. faecium* EN510 (PJVQ00000000) | D (191) | 2.46 | 38.1 | 2,405 | 158 | Chicken carcass |
| *E. faecium* LIT2 A36 (RKNA00000000) | D (108) | 2.52 | 38.1 | 2,502 | 181 | Litter |
| *E. faecium* P3 C A7 (RKQA00000000) | D (118) | 2.42 | 38.2 | 2,391 | 140 | Ceaca |
| *E. faecium* Efm33s (VFCU00000000) | D (116) | 2.61 | 38.1 | 2,554 | 116 | Blood |
| *E. faecium* ZGZA7-10 (JAJAGE000000000) | D (165) | 2.57 | 37.9 | 2,461 | 113 | Cheese |
| *E. faecium* 1001136B_160425_C7 (JADMUE000000000) | D (165) | 2.72 | 38 | 2,706 | 201 | Fecal |
| *E. faecium* HD5 (JADCMH000000000) | D (149) | 2.7 | 38 | 2,670 | 187 | Fecal |
| *E. faecium* UAA945 (AIUV00000000) | D (64) | 2.93 | 37.9 | 2,924 | 87 | Unknown |
| *E. faecium* UAA944 (AIUU00000000) | D (73) | 2.9 | 37.9 | 2,849 | 90 | Unknown |
| *E. faecium* AR98-565 (QPTK00000000) | D (159) | 2.84 | 37.8 | 2,824 | 83 | Drain fluid |
| *E. faecium* 9-F-6 (NMZI00000000) | D (6) | 3.15 | 37.7 | 3,090 | 161 | Fecal |
| *E. faecium* 71-1 (JAFLIT000000000) | D (58) | 2.63 | 38.1 | 2,570 | 119 | Probiotic |
| *E. faecium* M647h (JAMOBQ000000000) | D (56) | 2.61 | 38.2 | 2,532 | 114 | Meju |
| *E. faecium* BMECCcat (NMZK00000000) | D (2) | 3.35 | 38.2 | 3,234 | 122 | Fecal |
| *E. faecium* 7H8_DIV0219 (NGKW00000000) | D (123) | 3.26 | 37.8 | 3,268 | 167 | Fecal |
| *E. faecium* BSD2780061688st2_C8 (SPFM00000000) | D (265) | 3.19 | 37.7 | 3,175 | 91 | Tonsil crypt |
| *E. faecium* FDAARGOS_397 (PDEC00000000) | D (6) | 3.13 | 37.8 | 3,040 | 70 | Unknown |
| *E. faecium* UAA1280 (AJAD00000000) | D (50) | 3.12 | 38.3 | 2,958 | 74 | Fecal |
| *E. faecium* 1F1_DIV0518 (NGLS00000000) | D (33) | 3 | 38.1 | 2,922 | 124 | Human |
| *E. faecium* R.A73 (QOVC00000000) | D (28) | 2.94 | 38 | 2,817 | 58 | Mucus |
| *E. faecium* Hp_7-8 (PCFZ00000000) | D (175) | 2.89 | 38.2 | 2,955 | 143 | Fecal |
| *E. faecium* E1604 (AHXD000000000 | D (35) | 2.89 | 38.5 | 2,794 | 108 | Cheese |
| *E. faecium* FA3 (CP042834.1) | C (3) | 2.6 | 38.6 | 2,557 | 151 | Human |
| *E. faecium* 32-1 (JAFLIL000000000) | D (87) | 2.85 | 38 | 2,833 | 168 | Probiotic |
| *E. faecium* EF217 (JAIFOE000000000) | D (387) | 2.79 | 38.4 | 2,794 | 229 | Probiotic |
| *E. faecium* UC7256 (AWWM00000000) | D (143) | 2.82 | 38.1 | 2,773 | 171 | Food |
| *E. faecium* SN592 (VFCG00000000) | D (116) | 2.82 | 38 | 2,825 | 90 | Piggery air |
| *E. faecium* IIFCSG-B5 (JACGAP000000000) | D (71) | 2.82 | 38 | 2,812 | 171 | CRV environmental surface |
| *E. faecium* PC4.1 (ADMM00000000) | D (78) | 2.81 | 38 | 2,697 | 129 | Human |
| *E. faecium* 8S3 (PEID00000000) | D (91) | 2.81 | 38.2 | 2,764 | 170 | Milk |
| *E. faecium* E2620 (AHXW00000000) | D (26) | 2.81 | 38.3 | 2,725 | 138 | Blood |
| *E. faecium* UCN73 (JMIN00000000) | D (50) | 2.8 | 38.1 | 2,705 | 66 | Fecal |
| *E. faecium* KACC 15711 (LDND00000000) | D (147) | 2.78 | 38 | 2,798 | 144 | Meju |
| *E. faecium* K28_1 (JAASIF000000000) | D (99) | 2.78 | 38 | 2,662 | 128 | Fecal |
| *E. faecium* TX1330 (ACHL00000000) | D (156) | 2.78 | 38.3 | 2,663 | 82 | Human |
| *E. faecium* 4928STDY7071282 (LR607349) | D (247) | 2.77 | 40.2 | 2,359 | 71 | Fecal |
| *E. faecium* W141 (VHQS00000000) | D (82) | 2.76 | 38.2 | 2,732 | 175 | Beef carcass |
| *E. faecium* W148 (VHQR00000000) | D (96) | 2.76 | 38.2 | 2,745 | 167 | Beef carcass |
| *E. faecium* 11L2-121 (JAAMRV000000000) | D (57) | 2.76 | 38.2 | 2,700 | 124 | Chicken litter |
| *E. faecium* R2 (VHRJ00000000) | D (145) | 2.74 | 38.2 | 2,758 | 167 | Ground beef |
| *E. faecium* R4E (VHRB00000000) | D (100) | 2.74 | 38.2 | 2,745 | 175 | Ground beef |
| *E. faecium* CB_0179 (JABTDD000000000) | D (95) | 2.73 | 38.4 | 2,621 | 60 | Catch basin |
| *E. faecium* F1129D 110 (MJDY00000000) | D (182) | 2.71 | 38.3 | 2,710 | 156 | Fecal |
| *E. faecium* KACC 15962 (LDNF00000000) | D (102) | 2.72 | 38.2 | 2,719 | 132 | Doenjang |
| *E. faecium* L-X (JRGY00000000) | D (80) | 2.71 | 38.3 | 2,655 | 157 | Probiotic preparation |
| *E. faecium* E3083 (AHXZ00000000) | D (11) | 2.72 | 38.3 | 2,588 | 46 | Blood |
| *E. faecium* ENV-120 (JACZBO000000000) | D (80) | 2.7 | 38.2 | 2,656 | 127 | River |
| *E. faecium* KACC 15960 (LDNE00000000) | D (94) | 2.69 | 38.1 | 2,664 | 120 | Doenjang |
| *E. faecium* Com15 (CP025022) | D (335) | 2.7 | 40.7 | 2,208 | 90 | Unknown |
| *E. faecium* TX1337RF (AMAA00000000) | D (110) | 2.69 | 38.1 | 2,607 | 52 | Unknown |
| *E. faecium* ENV-269 (JACZBK000000000) | D (51) | 2.68 | 38.2 | 2,653 | 139 | River |
| *E. faecium* 4928STDY7071598 (CABGJZ000000000) | D (48) | 2.68 | 38.2 | 2,625 | 78 | Fecal |
| *E. faecium* CVM N55317 (PUAV00000000) | D (182) | 2.66 | 38.3 | 2,620 | 166 | Ground Beef |
| *E. faecium* KACC 16106 (LDNK00000000) | D (80) | 2.67 | 38 | 2,639 | 144 | Meju |
| *E. faecium* CVM N60190F (PTUC00000000) | D (115) | 2.66 | 38.3 | 2,602 | 67 | Young Chickens |
| *E. faecium* MJR8396B (LRPV00000000) | D (148) | 2.66 | 38.2 | 2,572 | 101 | Vagina |
| *E. faecium* CVM N59745F (PTUT00000000) | D (90) | 2.65 | 38.1 | 2,552 | 42 | Steers |
| *E. faecium* 1001095IJ_161003_B12 (JADMZV000000000) | D (55) | 2.65 | 38.3 | 2,584 | 134 | Fecal |
| *E. faecium* KACC 16097 (LDNI00000000) | D (65) | 2.63 | 38.2 | 2,579 | 122 | Meju |
| *E. faecium* 505 (AMBL00000000) | D (93) | 2.62 | 38.3 | 2,527 | 49 | Unknown |
| *E. faecium* KACC 16093 (LDNH00000000) | D (96) | 2.59 | 38.1 | 2,575 | 112 | Meju |
| ***E. lactis* KCTC 21015^T^ (CP065211)** | **C (1)** | **2.72** | **38.4** | **2,633** | **73** | **Raw milk cheese** |
| *E. lactis* 45-1 (JAFLIN000000000) | D (162) | 2.88 | 38 | 2,938 | 229 | Probiotic |
| *E. lactis* 46-1 (JAFLIO000000000) | D (146) | 2.88 | 38 | 2,938 | 225 | Probiotic |
| *E. lactis* DFI.7.70 (JAJDLB000000000) | D (69) | 2.88 | 38.2 | 2,858 | 128 | Fecal sample |
| *E. lactis* Hp_5-10 (PCGB00000000) | D (77) | 2.84 | 38.3 | 2,777 | 85 | Fecal |
| *E. lactis* KACC 16100 (LDNJ00000000) | D (105) | 2.84 | 38 | 2,817 | 150 | Meju |
| *E. lactis* CVM N59653F (PTTQ00000000) | D (109) | 2.83 | 38.1 | 2,783 | 87 | Sow |
| *E. lactis* 197EA1 (LEQR00000000) | D (10) | 2.84 | 38.2 | 2,767 | 81 | Breast meat |
| *E. lactis* XZ37302 (PGPO00000000) | D (143) | 2.81 | 38.2 | 2,766 | 126 | Fermented yak milk |
| *E. lactis* CICC 10840 (WOTQ00000000) | D (69) | 2.77 | 38.1 | 2,720 | 160 | Milk powder |
| *E. lactis* Tb32-6 (PGQJ00000000) | D (118) | 2.77 | 38.2 | 2,711 | 135 | Fermented yak milk |
| *E. lactis* 104-1 (JAFLJC000000000) | D (71) | 2.68 | 38.2 | 2,663 | 170 | Probiotic |
| *E. lactis* EF218 (JAIFOF000000000) | D (140) | 2.64 | 38.3 | 2,567 | 101 | Probiotic |
| *E. lactis* EF205 (JAIFNS000000000) | D (70) | 2.65 | 38.3 | 2,574 | 91 | Probiotic |
| *E. lactis* EF221 (JAIFOI000000000) | D (150) | 2.63 | 38.3 | 2,559 | 93 | Probiotic |
| *E. lactis* M212h (JAMOBR000000000) | D (7) | 2.74 | 38.3 | 2,697 | 81 | Meju |

^*^The genome analysis was conducted using the National Center for Biotechnology Information (NCBI) prokaryotic genome annotation pipeline (<http://www.ncbi.nlm.nih.gov/genome/annotation_prok/>).

^†^Genome status: D, draft; C, complete.

**Fig. S1** Heat-maps showing the pair-wise relatedness of 192 high-quality representative *E. faecium* and *E. lactis* genomes, based on average nucleotide identity (ANI) values. The strain names in GenBank are indicated as reported in the GenBank database and *E. lactis* strains that have been incorrectly annotated as *E. faecium* strains in GenBank are highlighted in red. The asterisks represent the type strains of *E. faecium* and *E. lactis*. The hierarchical clusters represented by dendrograms were constructed by simple linkage of the ANI values.

**
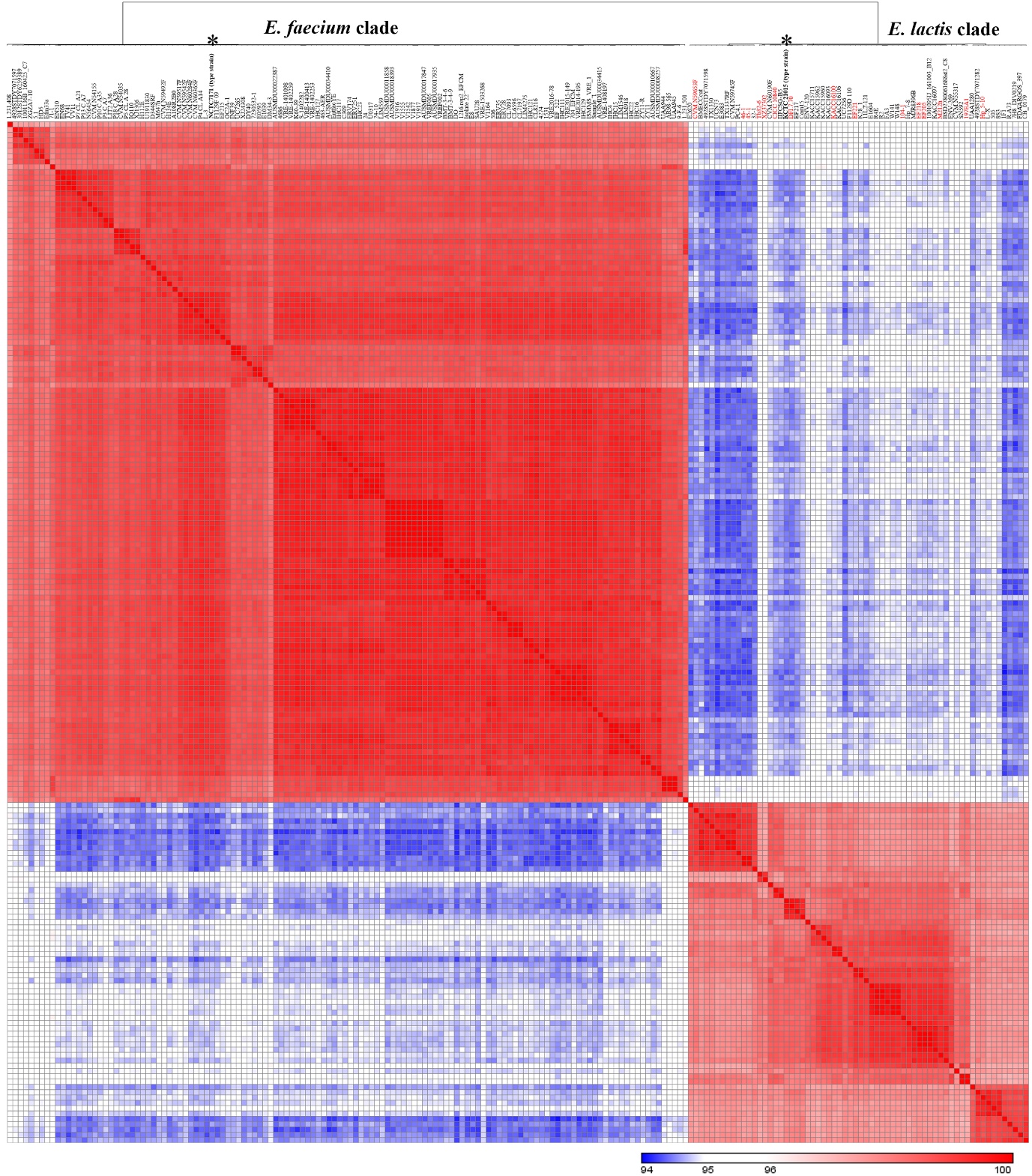
**

**Fig. S2** Phylogenetic tree of the *E. faecium* and *E. lactis* representative genomes using the maximum-likelihood algorithm, based on 16S rRNA gene sequences. The genomes of *E. faecium* and *E. lactis* strains that were classified by average nucleotide identity analysis in Fig. 1 are indicated using orange and blue letters, respectively. The type strains of *E. faecium* and *E. lactis* are indicated using an asterisk*.* *Enterococcus hirae* ATCC 9790^T^ (CP003504) was used as the out-group.

**
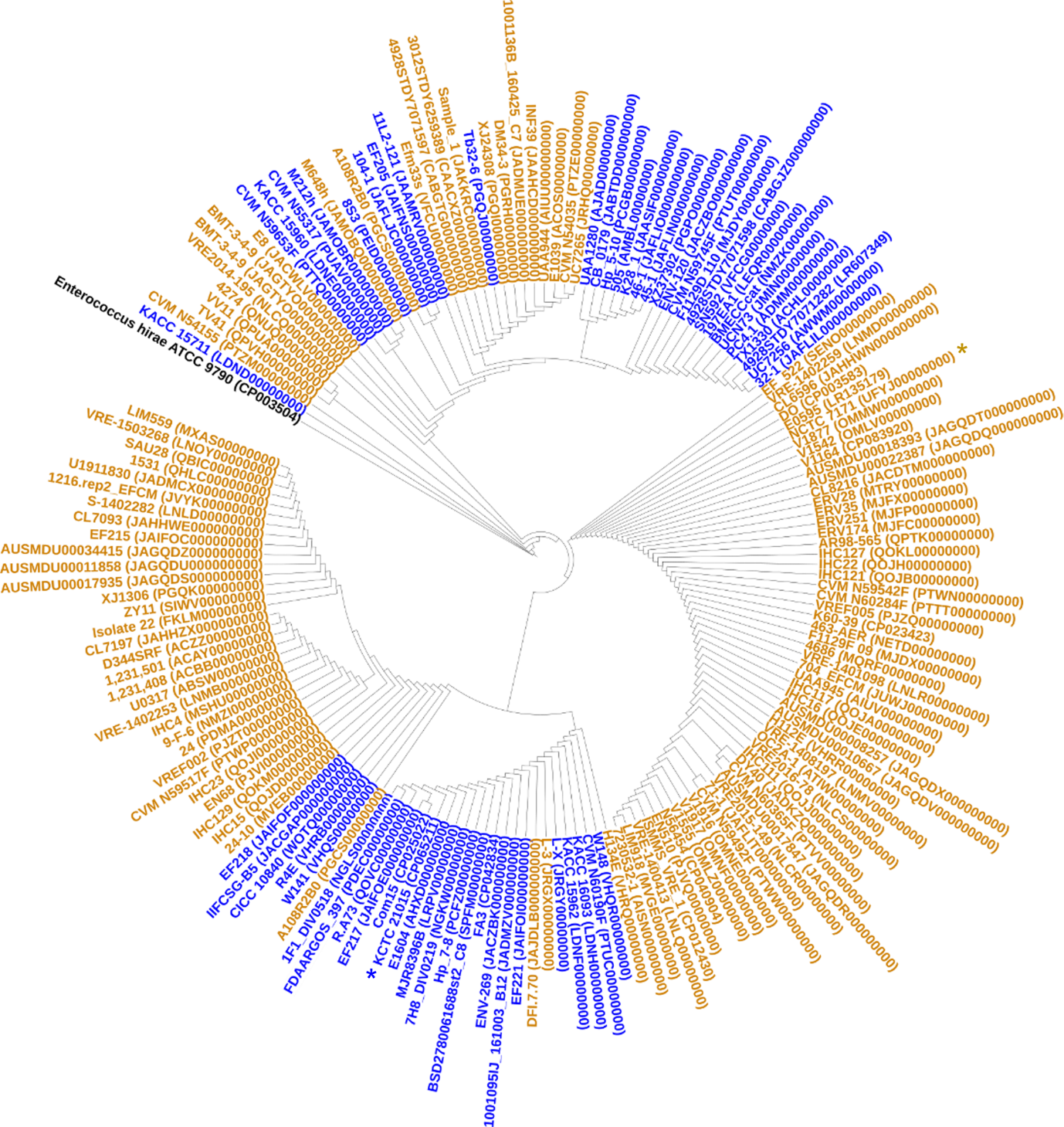
**

**Fig. S3** Relative abundances of KEGG functional genes identified in *E. faecium* and *E. lactis* genomes at the secondary (A) and tertiary (B) levels. The relative abundances are presented as percentages of genes assigned to their respective KEGG categories in relation to the total genes within each genome. The data are indicated as mean values of relative abundances in *E. faecium* and *E. lactis* genomes, with error bars representing standard deviations.


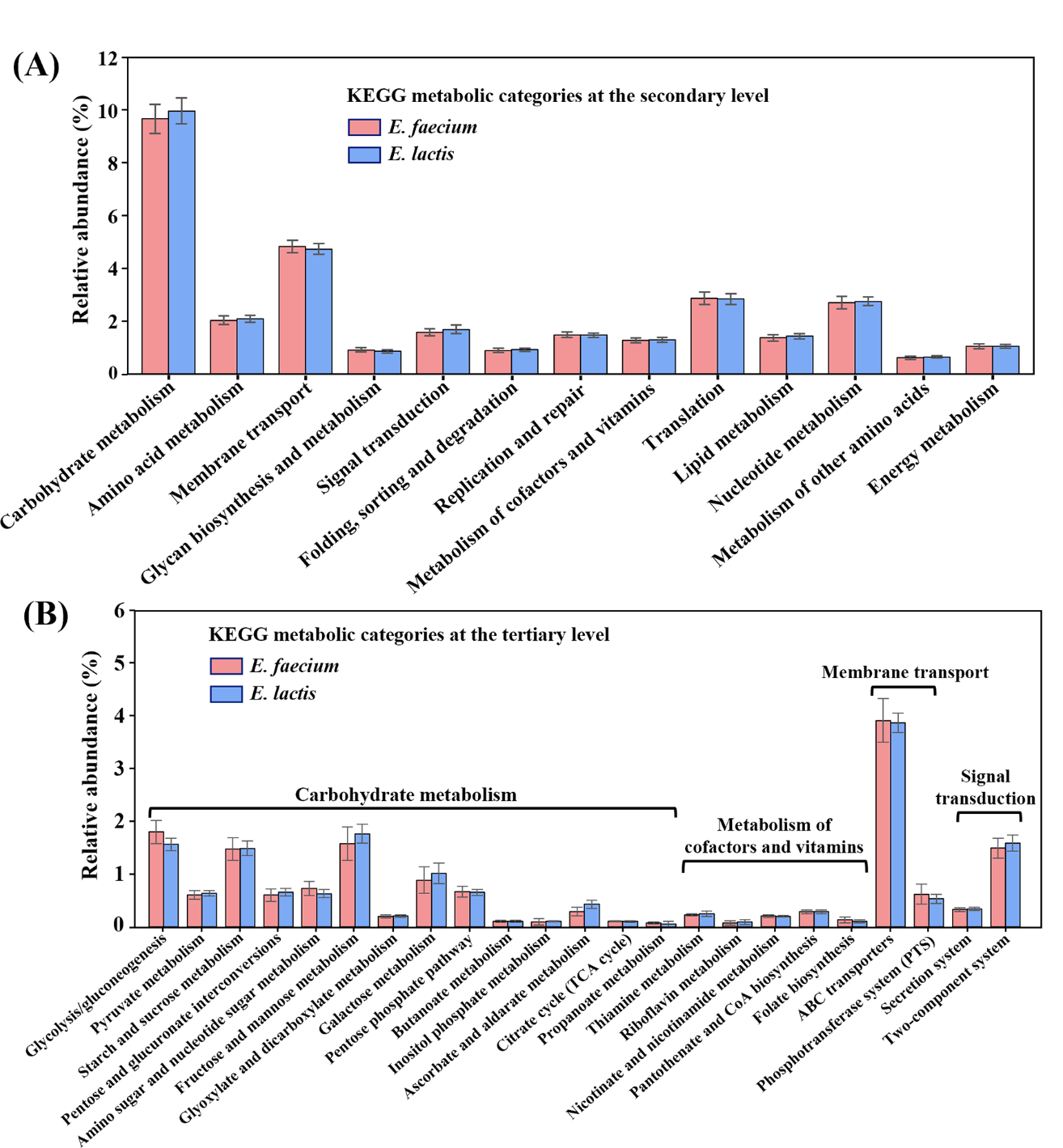


**Fig. S4** Proposed carbon metabolic pathways of the *E. faecium* and *E. lactis* species based on 128 representative genomes of *E. faecium* and 64 representative genomes of *E. lactis*. Metabolic pathways identified in over 97% of the representative genomes of both species, referred to as core pathways, are depicted in gray, and metabolic pathways found in only certain genomes of *E. faecium* and/or *E. lactis* are depicted in violet. The proportions (%) of genomes harboring the corresponding genes in *E. faecium* and *E. lactis* are indicated by red and blue numbers in parentheses, respectively. The dotted gray lines represent carbon-transporting systems or pathways that have not been identified but may be present in *E. faecium* and *E. lactis* strains.

**
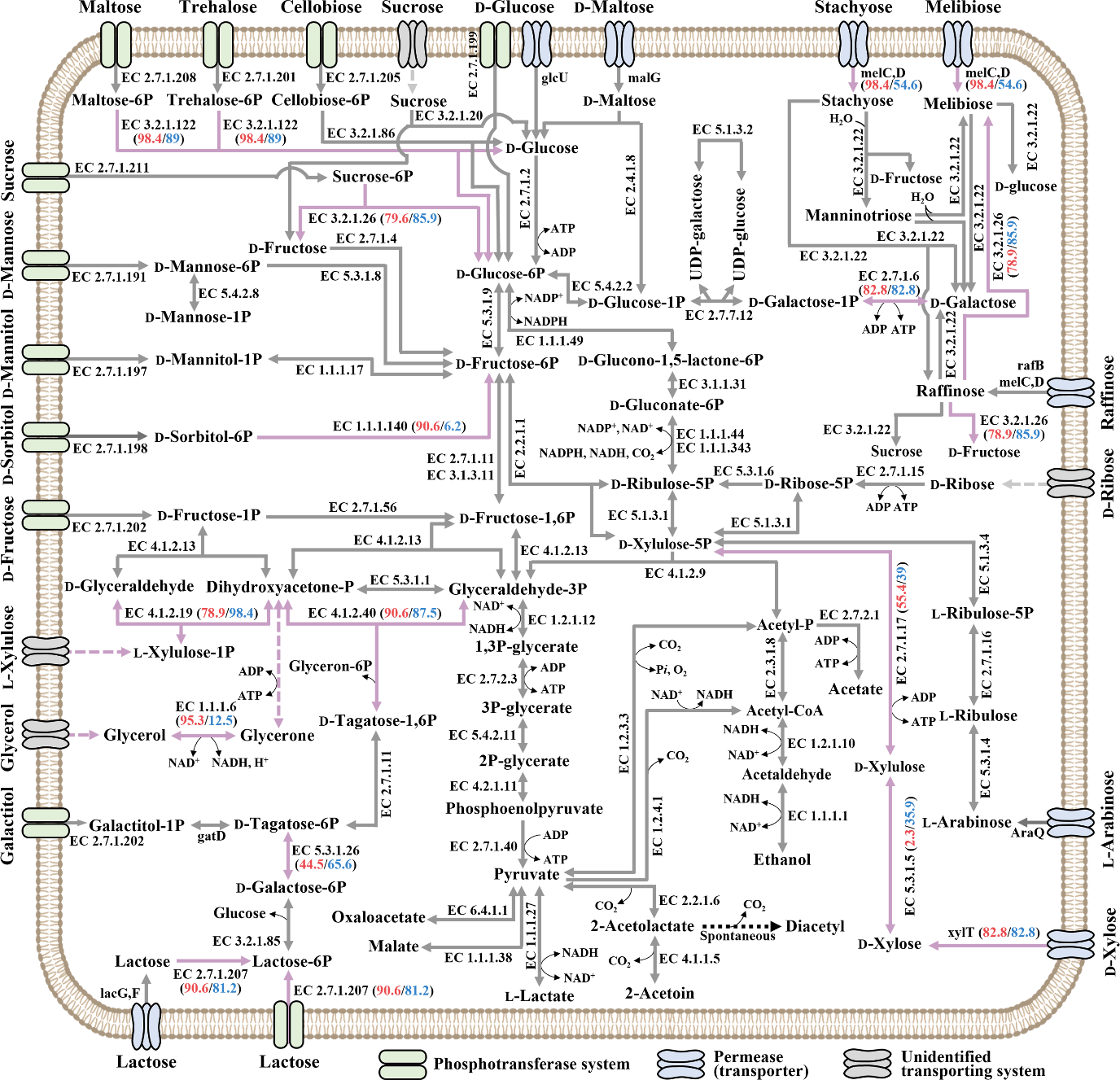
**

**Fig. S5** Box plots showing the gene abundances of five subcategories (A: transfer genes, B: integration/excision, C: stability/transfer/defense, D: replication/recombination/repair, and E: phage-specific genes) pertaining to mobile gene elements of Fig. 5D in *E. faecium* subclades I and II and *E. lactis* genomes. EF, *E. faecium*; EF-I, *E. faecium* subclade I; EF-II, *E. faecium* subclade II; EL, *E. lactis*. *, *p* < 0.01; **, *p* < 0.001; ***, *p* < 0.0001.


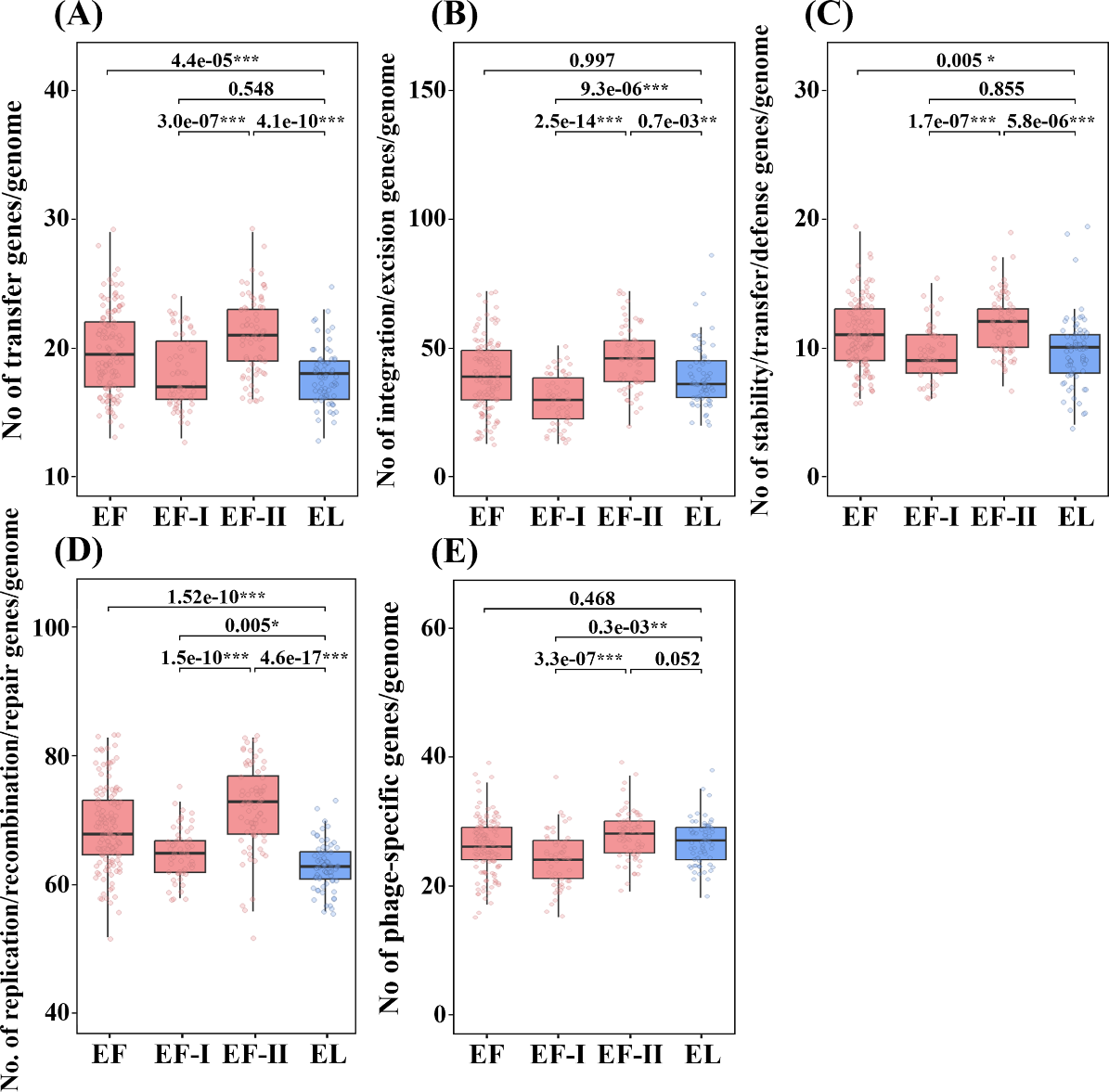


**Fig. S6** Phylogenetic trees of antibiotic resistance and virulence genes (not shown in Fig. 8), exhibiting differential abundances in the genomes of *E. faecium* (subclades I or II) and *E. lactis* by PCA. The trees were constructed using the maximum likelihood algorithm, based on the amino acid sequences. Distinct colors are assigned to *E. faecium* (subclades I or II) and *E. lactis*, corresponding to the source of gene sequences, which are displayed on the outer circle. Closely related GenBank sequences used as references are highlighted with a gray background. (A) *aphA*, aminoglycoside phosphotransferase; (B) *vanHA*, d-lactate dehydrogenase; (C) *vanSA*, histidine kinase; (D) *vanRA*, response regulator transcription factor; (E) *vanZA*, glycopeptide resistance protein; (F) *vanYA*, d-Ala-d-Ala carboxypeptidase; (G) *dfrF,* trimethoprim-resistant dihydrofolate reductase; and (H) *hylB*, hyaluronidase.


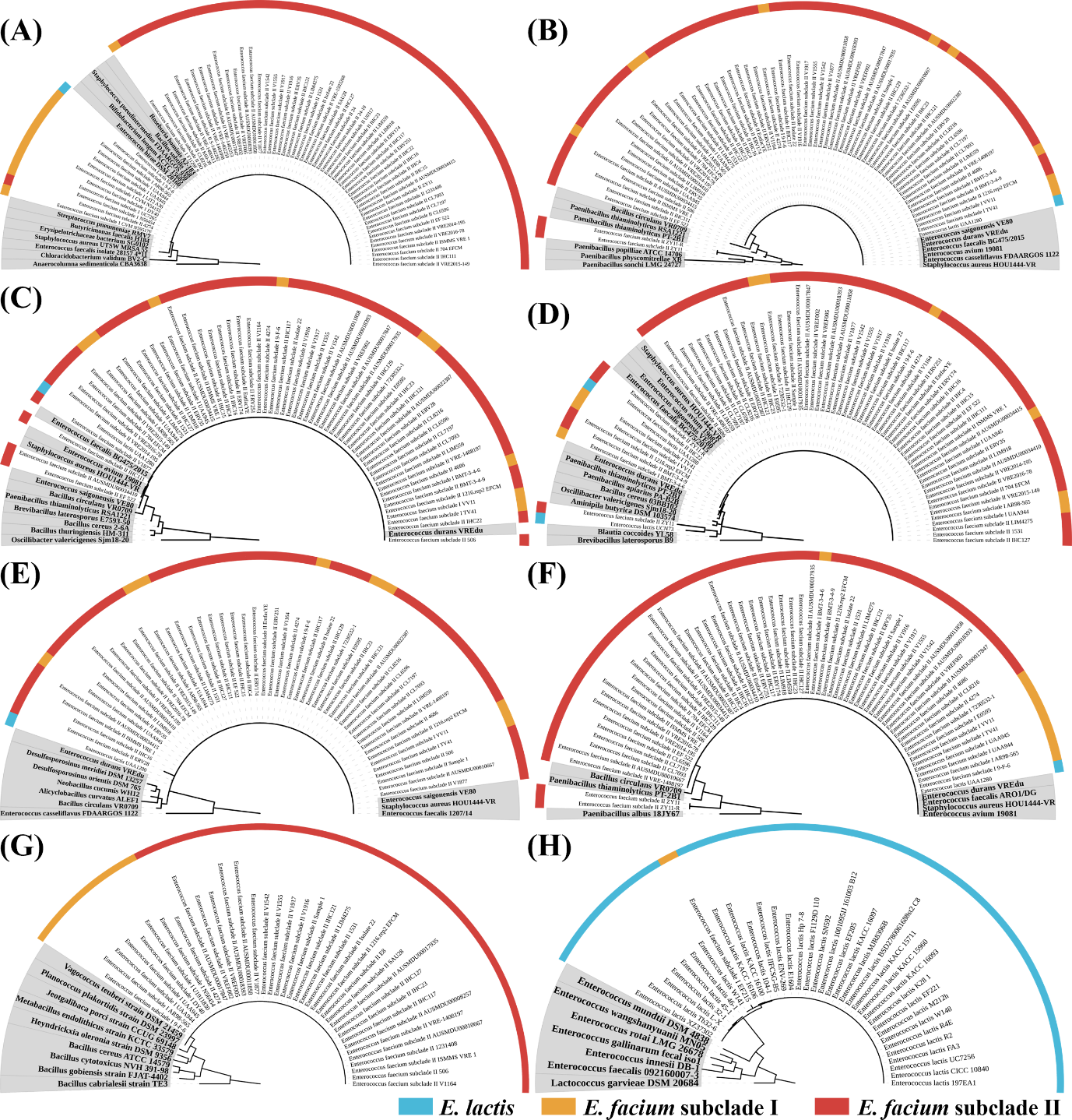

Supplement: Supplementary file 2 — Additional file 2. [file 12864_2023_9945_MOESM2_ESM.docx]
